# Bioinspired Polyacrylic Acid‐Based Dressing: Wet Adhesive, Self‐Healing, and Multi‐Biofunctional Coacervate Hydrogel Accelerates Wound Healing

**DOI:** 10.1002/advs.202207352

**Published:** 2023-04-14

**Authors:** Lingshuang Wang, Lian Duan, Ga Liu, Jianfeng Sun, Mohammad‐Ali Shahbazi, Subhas C. Kundu, Rui L. Reis, Bo Xiao, Xiao Yang

**Affiliations:** ^1^ State Key Laboratory of Silkworm Genome Biology College of Sericulture, Textile, and Biomass Sciences Southwest University Chongqing 400715 China; ^2^ Botnar Research Centre Nuffield Department of Orthopedics, Rheumatology, and Musculoskeletal Sciences University of Oxford Headington Oxford OX3 7LD UK; ^3^ Department of Biomedical Engineering University Medical Center Groningen University of Groningen Antonius Deusinglaan 1 Groningen 9713 AV Netherlands; ^4^ 3Bs Research Group I3Bs — Research Institute on Biomaterials, Biodegradables, and Biomimetics University of Minho Headquarters of the European Institute of Excellence on Tissue Engineering and Regenerative Medicine AvePark, Barco Guimaraes 4805‐017 Portugal

**Keywords:** antibacterial, polyacrylic acid, tannic acid, wet adhesion, wound healing

## Abstract

Polyacrylic acid (PAA) and its derivatives are commonly used as essential matrices in wound dressings, but their weak wet adhesion restricts the clinical application. To address this issue, a PAA‐based coacervate hydrogel with strong wet adhesion capability is fabricated through a facile mixture of PAA copolymers with isoprenyl oxy poly(ethylene glycol) ether and tannic acid (TA). The poly(ethylene glycol) segments on PAA prevent the electrostatic repulsion among the ionized carboxyl groups and absorbed TA to form coacervates. The absorbed TA provides solid adhesion to dry and wet substrates via multifarious interactions, which endows the coacervate with an adhesive strength to skin of 23.4 kPa and 70% adhesion underwater. This coacervate achieves desirable self‐healing and extensible properties suitable for frequently moving joints. These investigations prove that the coacervate has strong antibacterial activity, facilitates fibroblast migration, and modulates M1/M2 polarization of macrophages. In vivo hemorrhage experiments further confirm that the coacervate dramatically shortens the hemostatic time from hundreds to tens of seconds. In addition, full‐thickness skin defect experiments demonstrate that the coacervate achieves the best therapeutic effect by significantly promoting collagen deposition, angiogenesis, and epithelialization. These results demonstrate that a PAA‐based coacervate hydrogel is a promising wound dressing for medical translation.

## Graphical abstract

A polyacrylic acid (PAA)‐based coacervate hydrogel with robust wet adhesion and self‐healing capabilities is obtained through a mixture of PAA copolymers and tannic acid. This coacervate hydrogel promotes fibroblast migration, regulates macrophage polarization, kills bacteria, and facilitates wound healing via improving collagen deposition, angiogenesis, and re‐epithelialization, suggesting its considerable potential as a wound dressing.

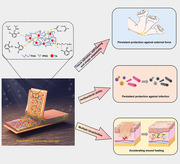

## 1 Introduction

The skin is the largest organ covering the underlying tissues and is vulnerable to injuries from external environmental forces.^[^ ^1^, ^2^ ^]^ Most common skin injuries self‐heal through several ordered stages, including hemostasis, inflammation, proliferation, and remodeling.^[^ ^3^, ^4^ ^]^ However, this sequential cutaneous healing can be easily disturbed by many detrimental factors, such as uncontrollable bleeding, bacterial infection, excessive inflammation, and secondary injuries.^[^ ^5^, ^6^, ^7^ ^]^ Therefore, advanced wound dressings with multiple biofunctions are urgently needed to assist in wound recovery.^[^ ^8^, ^9^, ^10^ ^]^

Polymeric materials are the most commonly used matrices for wound dressings. They can be processed into various forms, like films, hydrogels, and sponges, which can be loaded with bioactive ingredients to protect the wound and create a favorable environment for accelerating wound healing.^[^ ^11^, ^12^ ^]^ Polyacrylic acid (PAA) and its derivatives are FDA‐approved, low‐cost, nontoxic polymers with the capacity for dry adhesion and hemostasis,^[^ ^13^, ^14^ ^]^ and they have been widely applied in commercial wound dressings such as the 3M Nexcare bandage.^[^ ^15^ ^]^ Unfortunately, due to poor adhesion under moist conditions, the PAA‐based wound dressings often detach from the wound area because of exuding blood or other body fluids. The damp environment can disrupt the hydrogen bonding of adhesive groups and lower the surface energy of substrates, weakening or eliminating adhesion.^[^ ^16^ ^]^ Thus, scientists have struggled to develop materials with stronger wet adhesion. Wang et al.^[^ ^17^ ^]^ reported that PAA derivatives prepared from acrylic acid and 1‐vinylimidazole could robustly adhere to wet tissue via hydrogen bonds and electrostatic interactions. However, the materials are not widely used because of the toxicity of 1‐vinylimidazole and unsubstantiated biofunctions. Therefore, it is critical to develop novel PAA derivatives with multiple bioactivities that have the desired wet adhesion properties for wound dressings, but are safe.

Some aquatic organisms, such as sandcastle worms and mussels,^[^ ^18^, ^19^ ^]^ can secrete dopamine‐based proteins to firmly adhere to the sticky surface through multiple molecular interactions.^[^ ^20^, ^21^, ^22^ ^]^ Inspired by this, scientists have synthesized a variety of wet adhesives, among which coacervates containing tannic acid (TA) were the most promising because of their facile fabrication and efficient adhesion.^[^ ^23^, ^24^, ^25^, ^26^ ^]^ Given the beneficial bioactivities of TA,^[^ ^27^, ^28^ ^]^ these coacervates appear well‐suited for wound dressings. In theory, PAA‐based matrices could also be used to form coacervates with TA via hydrogen bonds because of the numerous carboxyl groups in the molecular chain.^[^ ^29^, ^30^ ^]^ The electrostatic repulsion between deprotonated carboxyl groups in a neutral solution prevented the aggregation of PAA chains and coacervate formation. While coacervates could be obtained by eliminating this repulsion in a solution with a relatively low pH of <1.5,^[^ ^31^ ^]^ this acidity would not be conducive to wound healing.

**Figure 1:**
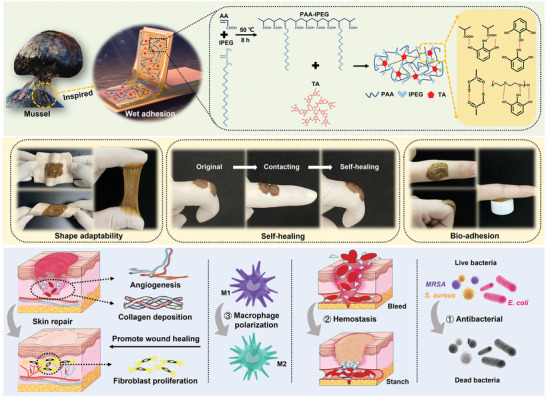
Illustration of the PAA‐IPEG/TA coacervate hydrogel endowed with robust wet tissue adhesion, shape adaptability, self‐healing, bio‐adhesion, antibacterial activity, hemostasis, and macrophage polarization regulation, which can promote collagen deposition, angiogenesis, and re‐epithelialization to accelerate wound closure and healing.

Thus, to develop PAA‐based wound dressings with multiple biofunctions and satisfactory wet adhesion, we utilized a simple one‐step reaction method to copolymerize nontoxic isoprenyl oxy poly(ethylene glycol) ether (IPEG) with acrylic acid. The fabricated copolymer consisting of a PAA backbone with short PEG branches is termed PAA‐IPEG. The oxygen atoms in the ether bonds of PEG segments can bind TA via hydrogen bonds.^[^ ^32^ ^]^ More importantly, the uncharged PEG segments have no repulsion to prevent the aggregation of molecular chains in the TA solution.^[^ ^33^, ^34^ ^]^ The Video S1, Supporting Information, shows that the PAA‐IPEG/TA coacervate hydrogel at neutral pH value is easily obtained. As seen in **Figure** **1**, the hydrogel is endowed by its large free catechol groups with the properties of wet adhesion, shape adaptability, self‐healing, and bioadhesion to adhere to moist wounds and provide long‐term protection against external forces and pathogenic bacteria. Also, the PAA‐IPEG/TA hydrogel can slowly and continuously release TA molecules to regulate macrophage polarization and promote wound healing through collagen deposition, angiogenesis, and re‐epithelialization.^[^ ^35^, ^36^, ^37^ ^]^

## 2 Results and Discussion

### 2.1 Construction and Physicochemical Characterization of Hydrogels

**Figure 2:**
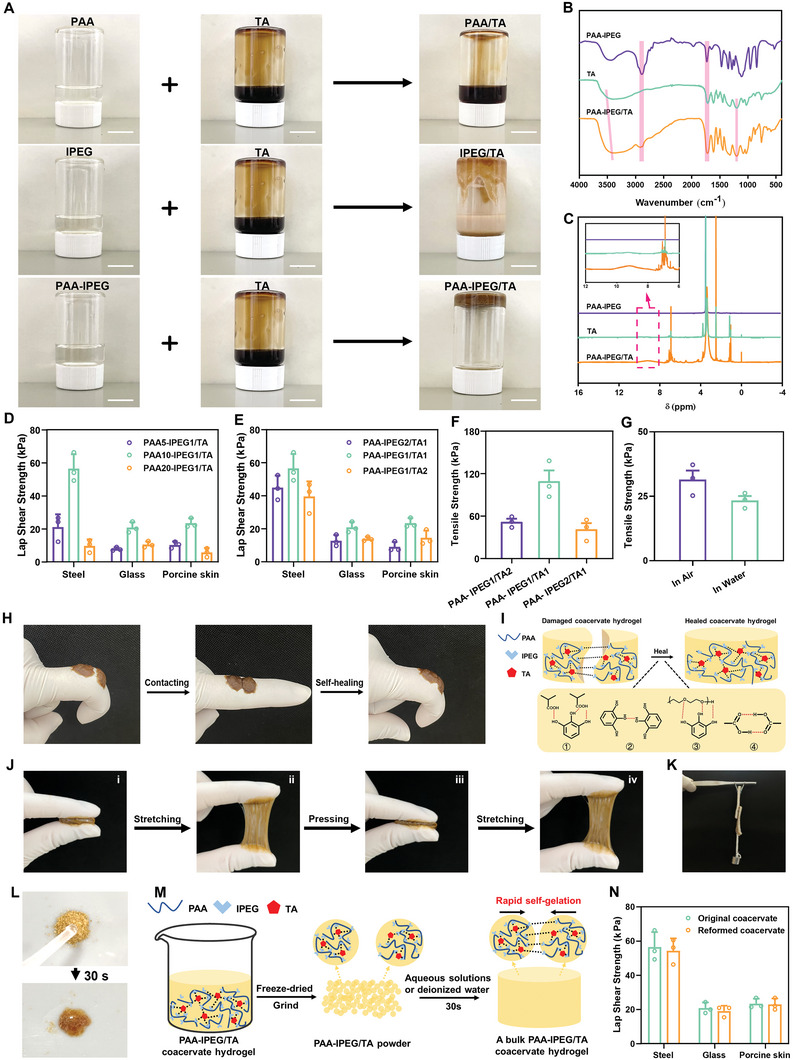
Physicochemical characterization and adhesion properties of the PAA‐IPEG/TA coacervate hydrogels. A) Gelling evaluation of the mixture of TA and PAA/IPEG/PAA‐IPEG by inverted tube tests. Scale bars = 1 cm. B,C) FTIR and ^1^H spectra of PAA‐IPEG/TA (orange), PAA‐IPEG copolymers (purple), and TA (green). D) Shear strength of coacervate hydrogels with different ratios of AA and IPEG (5:1, 10:1, and 20:1) adhering to various substrates (steel, glass, and porcine skin) by lap‐shear test (*n* = 3). E) Shear strength of coacervate hydrogels with different ratios of PAA‐IPEG copolymer to TA (2:1, 1:1, and 1:2) adhering to various substrates (steel, glass, and porcine skin) by lap‐shear test (*n* = 3). F) Tensile strength of the coacervate hydrogels with different ratios of PAA‐IPEG copolymers to TA (2:1, 1:1, and 1:2) adhering to PMMA by tensile test (*n* = 3). G) Tensile strength of the PAA‐IPEG/TA coacervate hydrogels adhering to PMMA in water and air by tensile test (*n* = 3). H) Photos showing self‐healing effects of PAA‐IPEG/TA coacervate hydrogels adhering to fingers. I) The self‐healing process of generating the PAA‐IPEG/TA coacervate hydrogels. J) Photos of the repeatedly stretched PAA‐IPEG/TA coacervate hydrogels: i) original state, ii) stretched, iii) recovered, and iv) restretched. K) Lap joint (bonded area: 1.5 cm × 1.5 cm) made of two pieces of porcine skin adhering to PAA‐IPEG/TA coacervate hydrogels, which could hold a mass of 50 g (≈0.49 N). L) Reformed PAA‐IPEG/TA coacervate hydrogels from PAA‐IPEG/TA powder added to water on PTFE plates. M) Illustration of reforming PAA‐IPEG/TA coacervate hydrogels from swelled powder. N) Comparison of adhesion strengths of freshly prepared PAA‐IPEG/TA coacervate hydrogels and reformed PAA‐IPEG/TA coacervate hydrogels on different substrates (steel, glass, and porcine skin, *n* = 3). Data are means ± S.E.M. (standard error of mean).

PAA is a common water‐soluble polymer, which has been extensively applied in commercial wound dressings.^[^ ^38^ ^]^ However, due to its weak wet adhesion, body fluids may cause it to detach from the wound. To address this issue, we developed a novel facile method inspired by the mussel's adhesion matrix and based on a mixture of PAA and TA. However, the PAA bound TA poorly because electrostatic repulsion among the ionized carboxyl groups restricted the aggregation of PAA chains and prevented the formation of coacervate hydrogel (**Figure** **2**A). To overcome this, we grafted electroneutral PEG segments onto PAA chains by free radical polymerization among acrylic acid and IPEG monomers, since IPEG strongly interacted with TA to form a coacervate hydrogel like melted chocolate (Figure 2A). However, the IPEG/TA was unsuitable for wound dressing because of insufficient mechanical properties to maintain its shape. The molecular weight of the obtained PAA‐IPEG was determined by gel permeation chromatography to be 570 kDa (Figure S1, Supporting Information). As shown in Figure 2A and Video S1, Supporting Information, by rapidly stirring a mixture of a PAA‐IPEG solution (20%, w/v) with a TA solution (50%, w/v), homogeneous coacervate hydrogels with a high level of wet adhesion were obtained, suggesting that the grafted PEG segments accelerated hydrogel formation.

The mechanism of PAA‐IPEG/TA coacervate hydrogel formation was determined by Fourier transform infrared spectroscopy (FTIR) and nuclear magnetic resonance (NMR). We theorized that PAA‐IPEG mainly interacted with the multiple aromatic ring structures of TA through hydrogen bonds.^[^ ^39^ ^]^ In the FTIR spectra (Figure 2B), the peak corresponding to the —OH stretching vibrations of the free catechol/pyrogallol groups in TA shifted from 3426 to 3402 cm^−1^. The peak related to the carbonyl bonds (C=O) in TA shifted from 1715 to 1725 cm^−1^ after the formation of the PAA‐IPEG/TA coacervate hydrogel. These shifts likely resulted from the changed vibrational energy of the O—H and C=O bonds after forming hydrogen bonds between PAA‐IPEG and TA.^[^ ^40^ ^]^ Furthermore, when PAA‐IPEG was cross‐linked with TA, the peaks corresponding to the C—H deformation vibrations (2888 cm^−1^) and C—O stretching vibrations (1192 cm^−1^) in the PAA‐IPEG molecular chains shifted to 2918 and 1198 cm^−1^, respectively, which also suggested the formation of hydrogen bonds between PAA‐IPEG and TA. The ^1^H NMR spectrum also evidenced the supramolecular architecture of the PAA‐IPEG/TA coacervate hydrogel. As shown in Figure 2C, the characteristic peaks of phenolic hydroxyl groups in TA were present at 8–10 ppm, while PAA‐IPEG exhibited no peaks in this range. In contrast, a broad peak was observed in the range from 8–10 ppm after the formation of PAA‐IPEG/TA coacervate hydrogels. To further validate the formation of hydrogen bonds between PAA‐IPEG and phenolic hydroxyl groups in TA, the PAA‐IPEG/TA coacervate hydrogel was treated with a concentrated urea solution (6 M) to break the hydrogen bonds.^[^ ^41^ ^]^ As a result, the PAA‐IPEG/TA coacervate hydrogel disintegrated after immersion in the 6 M urea solution for 24 h (Figure S2, Supporting Information). This phenomenon proved that the fabrication of the coacervate hydrogel relied on the formation of hydrogen bonds. Imaging with scanning electron microscopy (SEM) revealed that the interior (cross‐section) of the PAA‐IPEG/TA coacervate hydrogel had many irregular and uneven pores (Figure S3, Supporting Information).

### 2.2 Adhesion Performances of Hydrogels

As reported, polyphenols can provide strong adhesion in both dry and wet environments because of diverse molecular interactions with substrates.^[^ ^42^, ^43^ ^]^ We hypothesized that our PAA‐IPEG/TA coacervate hydrogel could robustly adhere to various materials. To validate this, several substrates, including metals, plastics, glass, ceramic, rubber, and porcine skin, were tested for the adhesion of the PAA‐IPEG/TA coacervate hydrogel in the air (Figure S4, Supporting Information). It was clear that the coacervate hydrogels firmly adhered to all these materials, regardless of the hydrophilicity of the surface. The adhesion was maintained for a long time, especially when the coacervate hydrogel was applied to a dry iron weight, even in water (bottom right corner of Figure S4, Supporting Information). We also investigated the wet adhesion capacities of the coacervate hydrogels with various substrates (Figure S5, Supporting Information). The PAA‐IPEG/TA coacervate hydrogel could easily adhere to these materials in water, even to polytetrafluoroethylene (PTFE), which is notorious for its low surface energy and strong resistance to adhesion. To further test the adhesion ability, a flow‐through method was implemented to simulate harsher wet conditions, as presented in Figure S6 and Video S2, Supporting Information. The coacervate hydrogels were adhered to a PTFE sheet and subsequently flushed with a continuous water flow. The results indicated that the coacervate hydrogels remained stably attached to the PTFE sheet after 1 h of flushing. A rotating disc experiment was also conducted to determine if the coacervate hydrogels would remain attached to porcine skin during aggressive stirring, which directly reflected the desired adhesive ability of the coacervate to a humid wound. Figure S7, Supporting Information, illustrates that there was no detachment from porcine skin even after 48 h of rapid stirring, thus proving the strong adhesive ability of the PAA‐IPEG/TA coacervate to a wet wound. To sum up, these experiments convincingly validated the robust and persistent adhesion of a PAA‐IPEG/TA coacervate hydrogel to diverse substrates, thus guaranteeing its potential utility in wound dressings.

Given that IPEG segments were crucial in forming the PAA‐IPEG/TA coacervate hydrogels, we quantitatively investigated the ratios of AA to IPEG in polymerization—for optimal adhesion performance. A series of PAA‐IPEG hydrogels with different IPEG contents were produced, and their adhesion strengths to various substrates were measured by lap shear strength testing (Figure 2D). The shear strengths of PAA10‐IPEG1/TA adhered on steel, glass, and porcine skin were 56.5, 20.8, and 23.4 kPa, respectively, which were significantly higher than those of PAA5‐IPEG1/TA (21.0, 10.5, and 10.3 KPa) and PAA20‐IPEG1/TA (9.7, 7.7, and 5.6 KPa), respectively. These observations suggested that the coacervate hydrogels made from PAA‐IPEG with 10% IPEG had the highest adhesion capacity, regardless of the substrate. PAA‐IPEG with lower IPEG content resulted in weak adhesion strength by decreasing TA absorption and the number of polyphenol groups in the coacervate hydrogels. PAA‐IPEG with excessively high IPEG contents can also lead to reduced adhesion strength due to the weakened interaction of the matrix by the plasticizing effect. The dynamic viscoelasticity of the coacervate with different IPEG ratios is shown in Figure S8, Supporting Information. The PAA10‐IPEG1/TA coacervate hydrogel had the most significant storage modulus (*G′*) compared with the other groups. It was reported that *G′* was directly related to the crosslinking density and stiffness of the network,^[^ ^44^, ^45^ ^]^ and our rheological results confirmed that the PAA10‐IPEG1/TA coacervate hydrogel had the highest crosslinking density. Figure S9, Supporting Information, illustrates the shear stress–strain curves of coacervate hydrogels with different IPEG ratios. Compared with other groups, PAA10‐IPEG1/TA coacervate hydrogel can withstand the higher strain, reaching 103%. Based on these experimental results, PAA10‐IPEG1 was selected as the copolymer in the follow‐up investigations.

Next, we evaluated the adhesion ability of the coacervate hydrogels with different amounts of PAA10/IPEG1 and TA. Figure 2E reveals that the shear strengths of PAA‐IPEG1/TA2, PAA‐IPEG1/TA1, and PAA‐IPEG2/TA1 when adhering to porcine skin were 14.4, 23.4, and 9.0 KPa, respectively. The coacervate hydrogels exhibited the maximum shear strength on glass and steel when the ratio of PAA‐IPEG to TA was 1:1, verified by testing tensile force (Figure 2F). After adhering to polymethyl methacrylate (PMMA) for 24 h, PAA‐IPEG1/TA1 exhibited a tensile strength of up to 109.3 ± 13.1 kPa, which was approximately twice as high as that of the other two mixtures. The explanation is that the low TA content decreased the level of free phenol hydroxyls that form the coacervate hydrogels at adhesion sites. An excess of TA would generate a stiff coacervate with redundant crosslinks and results in reduced adhesive ability. Rheological tests showed that the *G′* values of the coacervate hydrogels were positively proportional to the TA content, with high TA levels resulting in increased crosslink density (Figure S8, Supporting Information).

Underwater tensile adhesion tests showed that the PAA/IPEG‐TA coacervate had an average tensile strength of 23.4 kPa in water, which was ≈70% of that in the air adhesion test, indicating excellent underwater adhesive performance (Figure 2G). It is well known that the pH value is an essential factor affecting the adhesion ability of hydrogels.^[^ ^46^ ^]^ The wet adhesion strength of PAA‐IPEG/TA coacervate hydrogel under neutral and weak acid conditions (pH 5–7) was much higher than that under strong acid (pH 3) and alkaline conditions (pH 9–11) (Figure S10, Supporting Information). The results can be explained by the poor colloidal stability of PAA‐IPEG/TA induced by strong acids and oxidized polyphenolic structure of tannic acid in alkaline conditions, resulting in decreased adhesion.

In addition to the desirable adhesive ability in dry and wet conditions, the coacervate hydrogel possesses excellent self‐healing properties. To estimate the self‐healing capability of hydrogels, rheological thixotropy tests and macroscopic self‐healing tests were performed on PAA‐IPEG/TA coacervate hydrogels.^[^ ^47^ ^]^ Figure 2H shows two separated PAA‐IPEG coacervate hydrogels adhering to a person's bent knuckle. When the knuckle was straightened, the two separated coacervate hydrogels came into contact with each other and underwent self‐healing in 30 s. In the thixotropy experiments (Figure S11, Supporting Information), the *G′* value of coacervate hydrogel sharply decreased from 320 to 0.0005 Pa with an instantaneously increased strain from 1% to 400%, suggesting that the sizeable shear strain destroyed the network structure of coacervate hydrogel. When the loaded strain was removed, *G′* can instantly return to the original value, even after five alternate repetitive cycles, indicating that the destroyed network structure rapidly self‐healed via the dynamic hydrogen bonds. In practice, this self‐healing ability allows the PAA‐IPEG/TA coacervate hydrogel to protect the wound from external forces or physical movements that could cause a break in the wound dressing, potentially resulting in infection. As illustrated in Figure 2I, this property could be attributed to the rapid formation of dynamic hydrogen bonds on the contacting surface. Furthermore, we found that the PAA‐IPEG/TA coacervate hydrogel exhibited excellent extensibility (Figure 2J; Videos S3 and S4, Supporting Information) to withstand twisting when adhering to substrates (Figure S12, Supporting Information). In Figure S13, Supporting Information, the PAA‐IPEG/TA coacervate hydrogel was sufficiently flexible to remodel any complex 3D shape, including rabbits, stars, and Southwestern University logo, indicating that the coacervate hydrogel worked well in any shape and was well suited for body parts with frequent movements. Moreover, the hydrogel followed the shape of the container within 30 min, showing its excellent self‐adaptability (Figure S13 and Video S5, Supporting Information). As skin adhesion is essential for clinical wound dressings, the profile of the skin adhesion of the PAA‐IPEG/TA coacervate hydrogel was further assessed. As shown in Figure 2K, after adhering to the pigskin, the PAA‐IPEG/TA hydrogel could withstand the pull of a 50 g weight, a 200 g weight after adhering to metal, and a 500 g weight after an additional 2 h of aging (Figure S14, Supporting Information), proving sufficient adhesion for an effective wound dressing.

Although it showed good adhesion to various substrates, the as‐prepared PAA‐IPEG/TA coacervate hydrogel in the wet state is too adhesive to store and transport for clinical applications. The coacervate can be processed into a powder without adhesion by lyophilization. After rehydrating with water or phosphate buffer saline, the PAA‐IPEG/TA powders rapidly gel into adhesive coacervate hydrogels in bulk via dynamic hydrogen bonds (Figure 2L,M). Strikingly, our experiments revealed no difference between the reformed and the original coacervate hydrogels in terms of their adherence to various substrates, including steel, glass, and skin (Figure 2N). This proved that the powder was an ideal form for storage and transportation. Thus, the PAA‐IPEG/TA coacervate hydrogel could easily be scaled up for industrial production because of the low price of raw materials and the facile fabrication steps.

### 2.3 In Vitro Biocompatibility, Cell Migration, and Macrophage Polarization of Hydrogels

**Figure 3:**
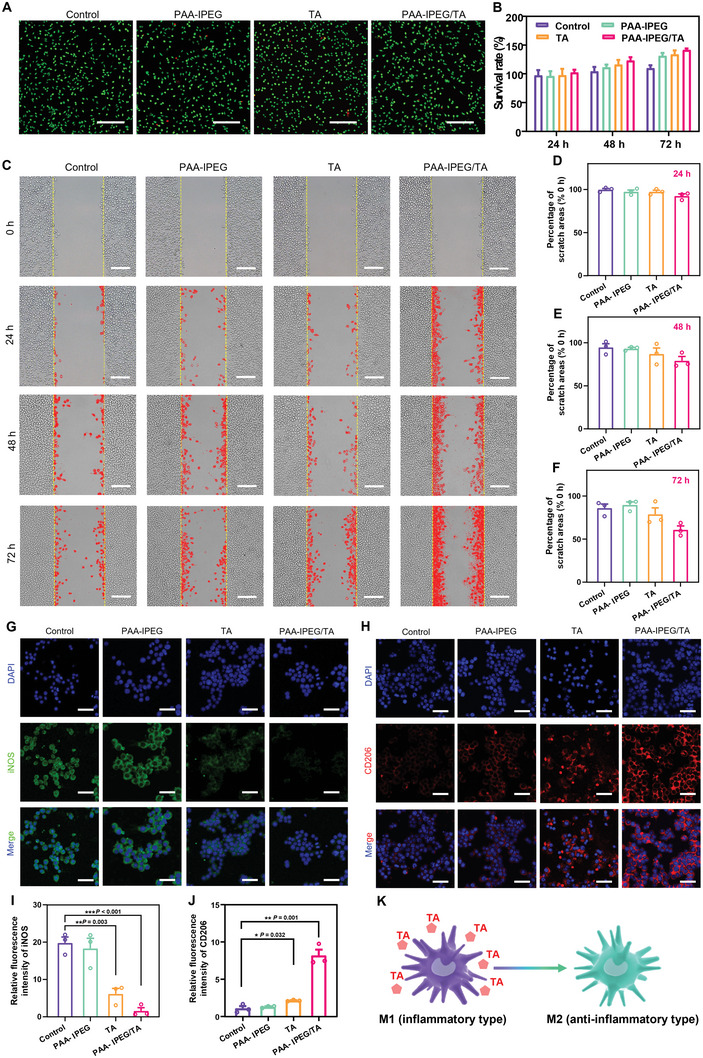
Effects of coacervate hydrogels on cytotoxicity, cell migration, and macrophage polarization. A) Live/dead staining of L929 cells after 24 h incubation with leach liquor of PAA‐IPEG copolymers, TA, or PAA‐IPEG/TA coacervate hydrogels. Scale bars = 200 µm. B) Cell survival percentage of L929 cells after 24, 48, and 72 h incubation with leach liquor of PAA‐IPEG copolymers, TA, or PAA‐IPEG/TA coacervate hydrogels (*n* = 5). C) Migration of L929 cells after incubation with leach liquor of PAA‐IPEG copolymers, TA, or PAA‐IPEG/TA coacervate hydrogels for 24, 48, and 72 h. Scale bars = 200 µm. D–F) Percentages of scratch areas of L929 cells after incubation with leach liquor of PAA‐IPEG copolymers, TA, or PAA‐IPEG/TA coacervate hydrogels for 24, 48, and 72 h (*n* = 3). G) Immunostaining of iNOS and H) CD206 in RAW 264.7 macrophages after incubation with leach liquor of PAA‐IPEG copolymers, TA, or PAA‐IPEG/TA coacervate hydrogels for 24 h. Scale bar = 50 µm. I) Quantification of relative fluorescence intensity of iNOS and J) CD206 using Image J (*n* = 3). K) Illustration of macrophage polarization regulation by slowly released TA from the PAA‐IPEG/TA coacervate hydrogels. Statistical significance was expressed as *p < 0.05, **p < 0.01, and ***p < 0.001. Data are means ± S.E.M. (standard error of the mean).

Since biocompatibility is a prerequisite for the clinical translation of biomedical products, this property of the PAA‐IPEG/TA coacervate hydrogel was evaluated using live/dead staining and 3‐(4,5‐dimethylthiazol‐2‐yl)‐2,5‐diphenyltetrazolium bromide (MTT) assays.^[^ ^48^, ^49^ ^]^ The fluorescence images in **Figure** **3**A revealed that most of the L929 cells from all the groups were stained green (live cells), and very few cells were stained red (dead cells), which was in agreement with the quantitative results (Figure S15, Supporting Information). The MTT data revealed that all the treatment groups achieved cell viabilities of >90%, suggesting that, as constructed, coacervate hydrogels possessed satisfactory biocompatibility for biomedical application (Figure 3B).

To investigate the wound healing effects of various samples, a cell monolayer scratch assay was used to simulate the wound‐healing process.^[^ ^50^ ^]^ After L929 cells had grown to confluence, a scratch was made with a sterile pipet tip across the monolayer. The regrowth of the “wounded” areas was measured as a function of time from 0 to 72 h. We observed that the L929 cells in the PAA‐IPEG/TA‐treated group migrated much faster into the scratched area compared with the other treatment groups (Figure 3C). The semi‐quantitative results of the scratch assay (Figure 3D–F) showed that the “wounded” area in the PAA‐IPEG/TA‐treated group had regrown by around 40% after co‐incubation for 72 h. These findings might be attributed to the pro‐migratory and mitogenic effects of the constantly released TA from the PAA‐IPEG/TA hydrogels.

Macrophages play an essential role in tissue repair and remodeling, and they can be polarized into M1‐type and M2‐type macrophages.^[^ ^51^ ^]^ It is known that M1 macrophages participate in pro‐inflammatory responses, which are essential in host defense against bacterial and viral infection. In contrast, M2 macrophages are associated with anti‐inflammatory reactions, tissue remodeling, and fibrosis.^[^ ^52^, ^53^ ^]^ Hence, the polarization‐promoting effect of hydrogel samples was examined using the macrophage cell line, RAW 264.7.^[^ ^54^ ^]^ As presented in Figure 3G–J, after incubation with different hydrogels for 24 h, there was no statistically significant difference in the polarization capacity between the control group and the PAA‐IPEG‐treated group. Only a small percentage of macrophages were polarized into M2‐type upon TA treatment. The PAA‐IPEG/TA‐treated macrophages exhibited the weakest green fluorescence (Figure 3G), but the most robust red fluorescence (Figure 3H) among all the groups, which was consistent with the fluorescence intensity profiles of the various treatment groups (Figure 3I,J). These results demonstrate that the coordination of PAA‐IPEG and TA can effectively promote the polarization of macrophages from M1‐ to M2‐type (Figure 3K), implying the potential to reduce inflammatory responses, remodel granulation tissue, and repair wounds.

### 2.4 In Vitro and Vivo Antibacterial Activity of Hydrogels

**Figure 4:**
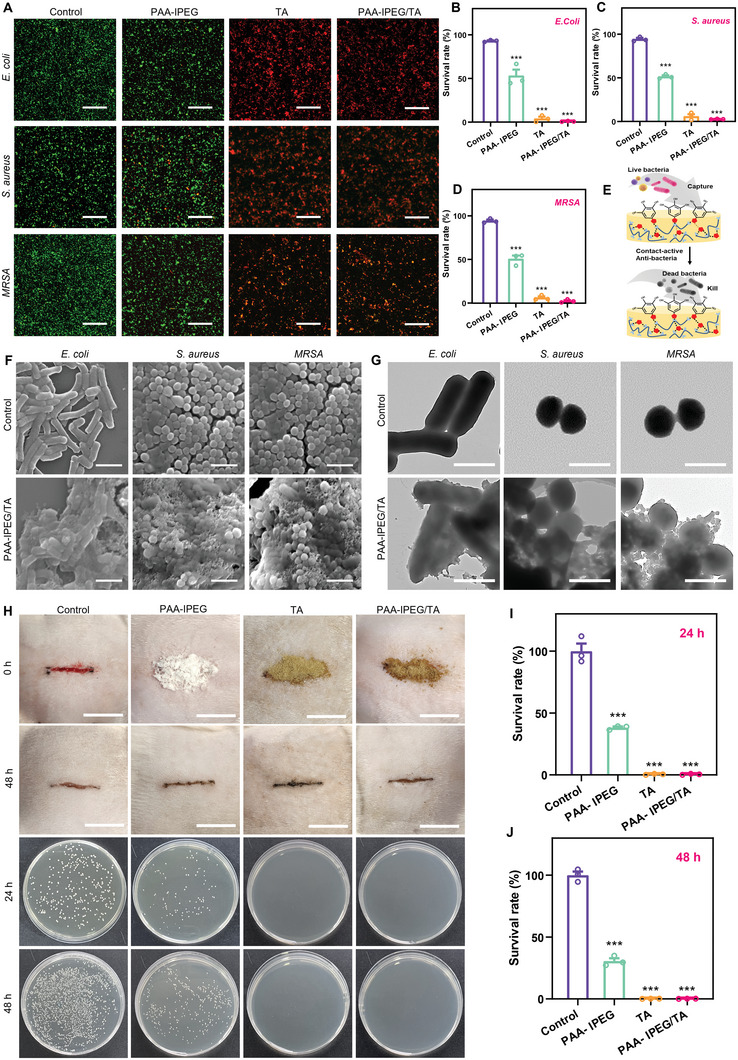
Antibacterial activities of PAA‐IPEG/TA coacervate hydrogels. A) Live/dead staining of *E. coli, S. aureus*, and MRSA after 6 h incubation with PAA‐IPEG copolymers, TA, or PAA‐IPEG/TA coacervate hydrogels. Scale bar = 100 µm. B–D) Survival rates of *E. coli*, *S. aureus*, and MRSA after 6 h incubation with PAA‐IPEG copolymers, TA, or PAA‐IPEG/TA coacervate hydrogels (*n* = 3). E) Diagram showing antibacterial activity of polyphenols in PAA‐IPEG/TA coacervate hydrogels. F) SEM images of *E. coli*, *S. aureus*, and MRSA after incubation with the PAA‐IPEG copolymers, TA, or the PAA‐IPEG/TA coacervate hydrogels for 6 h. Scale bar = 2 µm. G) TEM images of *E. coli*, *S. aureus*, and MRSA after incubation with PAA‐IPEG copolymers, TA, or PAA‐IPEG/TA coacervate hydrogels for 6 h. Scale bar = 1 µm. H) Photographs of infected full‐thickness rat dorsal skin wounds at 0 and 48 h with different treatments and colonies of MRSA bacteria surviving on agar plates of rat subcutaneous tissue under different treatment conditions. Scale bar = 1.5 cm. I,J) At 24 and 48 h, the quantitative bacterial survival rate of MRSA was extracted from control, PAA‐IPEG, TA, and PAA‐IPEG/TA treated groups (*n* = 3). Statistical significance was expressed as *p < 0.05, **p < 0.01, and ***p < 0.001. Data are means ± S.E.M. (standard error of the mean).

An unclosed wound without skin protection or a bandage is vulnerable to infection with pathogenic bacteria because of the direct exposure of subcutaneous tissue to the external environment.^[^ ^55^ ^]^ Bacterial invasion into the wound induces not only the degradation of healthy tissue but also continuous inflammation, which delays wound healing and can even cause complications like sepsis.^[^ ^56^, ^57^ ^]^ Accordingly, wound dressings like the hydrogels should possess effective antibacterial properties. Accumulating evidence has demonstrated that TA can efficiently kill Gram‐negative and Gram‐positive bacteria by disrupting the expression of functional proteins in bacterial cell walls and cytomembranes.^[^ ^58^, ^59^ ^]^ This suggests that the PAA‐IPEG/TA coacervate hydrogel might also have antibacterial activity. To test this supposition, bacteriostatic experiments against *Escherichia coli* (*E. coli)*, *Staphylococcus aureus* (*S. aureus)*, and methicillin‐resistant *Staphylococcus aureus* (MRSA) were performed using a live/dead dual‐fluorescence staining assay. As shown in **Figure** **4**A, bacteria in the control group and the PAA‐IPEG‐treated group exhibited predominantly green fluorescence (live bacteria), but only sparse red fluorescence (dead bacteria). In contrast, green fluorescent signals were sparingly dispersed in the TA‐treated and the PAA‐IPEG/TA‐treated groups, suggesting that TA and PAA‐IPEG/TA had substantial antibacterial activities against both susceptible and multi‐drug resistant bacteria. These observations aligned with the corresponding quantitative results that PAA‐IPEG killed almost 0% of bacteria but TA and PAA‐IPEG/TA killed more than 99.0% of bacteria within 6 h (Figure 4B–D). The results indicate that the antibacterial capacity of the PAA‐IPEG/TA coacervate hydrogel originates from TA molecules. In addition, colony counting on agar plates was performed to further validate the antibacterial activity of the various treatment groups (Figure S16, Supporting Information). The results confirmed the vital capacity of TA and PAA‐IPEG/TA to eliminate *E. coli*, *S. aureus*, and MRSA.

To determine the antibacterial mechanism of the PAA‐IPEG/TA coacervate hydrogels, the morphological features of bacteria exposed to coacervate hydrogels were investigated by SEM and transmission electron microscopy (TEM) (Figure 4F,G).^[^ ^60^ ^]^ The untreated *E. coli*, *S. aureus*, and MRSA cells in the control group were intact with distinct, smooth surfaces. In contrast, most of the bacteria on the surface of the PAA‐IPEG/TA coacervate hydrogel collapsed with a fused cytoderm, suggesting a high proportion of dead bacteria. Furthermore, the bright‐field TEM images (Figure 4G) showed that the areas of all three types of bacteria in the PAA‐IPEG/TA‐treated group became more transparent than those in the control group, indicating decreased bacterial contents. Thus, the antibacterial mechanism might involve TA molecules in the PAA‐IPEG/TA coacervate hydrogel that capture the bacteria and break the cytoderm, resulting in leakage of the bacterial contents and death (Figure 4E). These promising results support the idea that the PAA‐IPEG/TA coacervate hydrogel has active antibacterial properties that could protect a wound during healing.

The in vivo antibacterial effect of PAA‐IPEG/TA coacervate hydrogel has been verified via MRSA‐infected incisions on the back of rats. After 48 h of treatment, slight subcutaneous abscesses were observed in the control group, while PAA‐IPEG/TA coacervate hydrogel‐treated group exhibited smooth wounds without symptoms of infection (Figure 4H). Subsequently, the subcutaneous tissue of the injury was harvested, homogenized, and cultured on agar plates for standard plate counts after treatment for 24 and 48 h. The photographs show that no bacterial colony was observed in the TA and PAA‐IPEG/TA coacervate hydrogel‐treated groups (Figure 4H). The corresponding quantitative analysis suggested that CFU counts were reduced by more than 99% compared to the control and PAA‐IPEG groups (Figure 4I,J). These results demonstrated that PAA‐IPEG/TA coacervate hydrogel had an excellent antibacterial ability to prevent wound infection.

### 2.5 Blood Clotting and Hemostasis of Hydrogels

**Figure 5:**
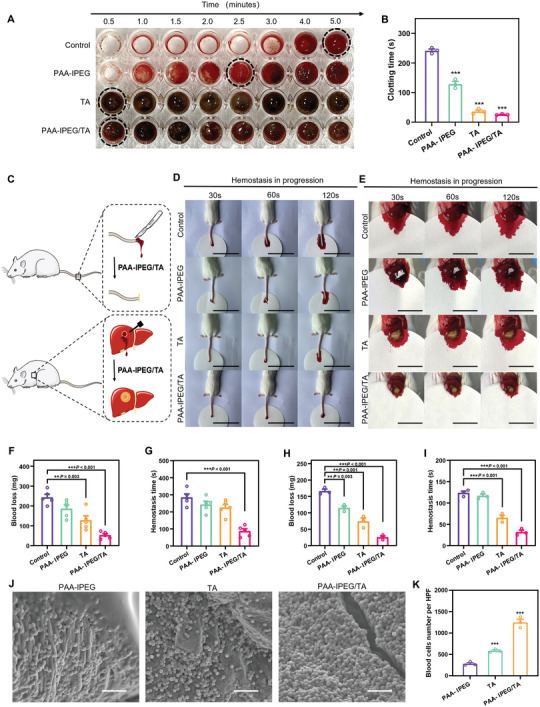
Hemostasis activity of PAA‐IPEG/TA coacervate hydrogels. A) Photographs of clotting after adding different hemostatic materials. B) Corresponding clotting time (*n* = 3). C) Illustration of protocols for the hemostatic experiments on two mouse models, tail amputation, and liver hemorrhage). Photos of hemostatic progression at D) the amputated tail (Scale bar = 6 cm) and on E) the hemorrhagic liver (Scale bar = 2 cm) treated with PAA‐IPEG copolymers, TA, and PAA‐IPEG/TA coacervate hydrogel powders, respectively. F) Blood loss and G) hemostatic time on the mouse tail amputation model (*n* = 5). H) Blood loss and I) hemostatic time on the mouse liver hemorrhage model (*n* = 3). J) SEM images of blood cells on the surface of the PAA‐IPEG copolymers, TA, and PAA‐IPEG/TA coacervate hydrogels. Scale bar = 20 µm. K) Quantification of blood cell adhesion on the surface of the PAA‐IPEG copolymers, TA, and PAA‐IPEG/TA coacervate hydrogels in a high‐power field (HPF, original magnification × 800, *n* = 3). Statistical significance was expressed as *p < 0.05, **p < 0.01, and ***p < 0.001. Data are means ± S.E.M. (standard error of the mean).

In most cases, bleeding can be inhibited through the synergistic effect of vasoconstriction, platelet aggregation, and blood coagulation.^[^ ^61^ ^]^ Nevertheless, hemostasis still requires wound dressings because some irregular damages, such as serious artery injuries, can cause uncontrollable hemorrhage and delay wound healing.^[^ ^62^, ^63^ ^]^ We hypothesized that the flexible shape, strong self‐healing capability, and outstanding adhesion of the PAA‐IPEG/TA coacervate hydrogel would be an ideal fit and adhere to an irregular wound to stop bleeding. In addition, the strong water absorption of PAA polymers and the efficient blood protein absorption of TA could endow the coacervate hydrogels with the ability to accelerate blood coagulation. Therefore, we designed a series of experiments to test their hemostatic capacity. In an initial investigation, droplets of blood from heparinized mice were converted into a gel within a few seconds of the addition of solid PAA‐IPEG/TA (Figure S17, Supporting Information). We also found that the blood‐PAA‐IPEG/TA coacervate hydrogel firmly adhered to the tube wall against the pull of gravity (Figure S18, Supporting Information), implying that the PAA‐IPEG/TA could fuse with blood to accelerate blood coagulation. In contrast, blood mixed with solid PAA‐IPEG or TA remained free‐flowing down the tube wall, indicating that neither powder alone formed a gel. Subsequently, PAA‐IPEG, TA, and PAA‐IPEG/TA were added to the whole blood to measure the clotting time. As shown in **Figure** **5**A,B, the clotting time significantly decreased from 241.7 to 25.7 s after being treated by PAA‐IPEG/TA. The results demonstrated that PAA‐IPEG/TA had the excellent clotting ability and can dramatically shorten the clotting time.

To evaluate the hemostatic potential of PAA‐IPEG/TA, an experiment illustrated in Figure 5C was performed using two hemorrhagic models of amputated mouse tail and damaged mouse liver, respectively. The blood diffusion rate on filter paper was taken as an evaluation metric to determine hemostasis. Notably, the filter paper in the PAA‐IPEG/TA‐treated group showed the least blood diffusion in both hemorrhagic models (Figure 5D,E), suggesting that PAA‐IPEG/TA could rapidly form a blood clot to stop bleeding after adhering to the wound. The hemostatic ability was also quantified as blood loss per unit time (30 s) and hemostatic time (Figure 5F–I). The results showed that the average blood loss of the PAA‐IPEG/TA‐treated group were 52.3 and 25.2 mg for the tail and liver models, respectively, which were significantly lower than those of the control group (242.9 and 165.7 mg), the PAA‐IPEG‐treated group (185.7 and 114.7 mg), and the TA‐treated group (127.6 and 73.7 mg). Also, the PAA‐IPEG/TA‐treated group had the lowest blood loss per unit time during the hemostatic process (Figure S19, Supporting Information). The hemostatic time was also notably shortened with the PAA‐IPEG/TA treatment. The PAA‐IPEG/TA powders could stop bleeding within 87.0 and 31.7 s for both experiments (tail and liver), respectively. The hemostatic time of the PAA‐IPEG‐treated group (242.0 and 116.7 s) and the TA‐treated group (224.0 and 65.0 s) was also much lower than that of the control group (280.0 and 123.3 s). The data demonstrate that PAA‐IPEG and TA favor blood coagulation, thus accelerating the hemostasis of the PAA‐IPEG/TA coacervate hydrogels.

SEM images of blood clots (Figure 5J) and the corresponding quantitative results (Figure 5K) revealed that the number of red blood cells adhering to the coacervate hydrogels was much higher than that in other groups. In all, the PAA‐IPEG/TA coacervate hydrogel has excellent blood absorption and blood coagulation abilities due to a synergistic effect of the interconnected porous microstructures (Figure S3, Supporting Information), the efficient blood protein absorption ability of TA, and the hydrophilic carboxyl groups on PAA segments.

### 2.6 In Vivo Wound Healing Performance of Hydrogel

The healing of a skin wound is usually divided into four continuous and coordinated steps: hemostasis, inflammation, proliferation, and remodeling.^[^ ^64^, ^65^, ^66^ ^]^ We hypothesize that the PAA‐IPEG/TA coacervate hydrogel accelerates wound healing through the substantiated hemostasis, promotion of cell migration, anti‐inflammation, and cell regulation abilities.

**Figure 6:**
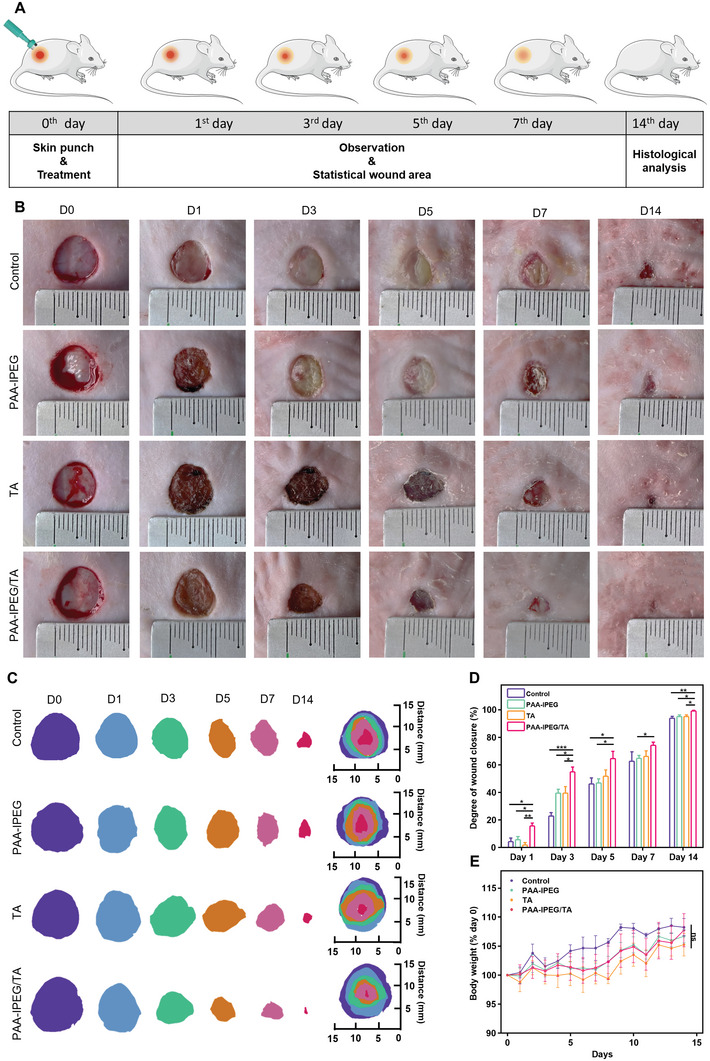
In vivo wound healing performance of coacervate hydrogels. A) Protocol of the wound healing experiment on the full‐thickness skin model. B) Representative photographs of skin wounds treated with Tegaderm film (control), PAA‐IPEG copolymers, TA, and PAA‐IPEG/TA coacervate hydrogels on days 0, 1, 3, 5, 7, and 14. C) Traces of wound closure on days 0, 1, 3, 5, 7, and 14. D) Wound closure percentages. E) Body weights. Statistical significance was expressed as *p < 0.05, **p < 0.01, and ***p < 0.001. Data are means ± S.E.M. (standard error of the mean).

Full‐thickness skin wound experiments were performed on rats to determine the therapeutic effects of the PAA‐IPEG/TA coacervate hydrogel (**Figure** **6**A). A wound with a diameter of 10 mm was produced on the dorsal skin, and the healing rate was recorded during the following 14 days (Figures 6B,C). We found that the wound area in each group gradually decreased over time. Wounds in the PAA‐IPEG/TA‐treated group were almost completely healed by day 14, while those in the other groups took longer. As seen in Figure 6D, the percentages of wound closure in the control group, the PAA‐IPEG‐treated group, the TA‐treated group, and the PAA‐IPEG/TA‐treated group were 22.8%, 39.5%, 39.5%, and 54.8% on day 3 at the early stage of wound recovery, subsequently increased to 62.5%, 64.7%, 66.1%, and 74.6% on day 7, and finally reached 93.8%, 95.1%, 94.9%, and 99.0% on day 14, respectively. These data show that the best therapeutic effect was achieved by PAA‐IPEG/TA, followed by PAA‐IPEG and TA. Therefore, we speculate that PAA‐IPEG and TA perhaps had a synergistic effect in promoting the therapeutic activity of PAA‐IPEG/TA. Additionally, we observed no significant difference in body weight (an important indicator for potential toxicity of medical devices) between the three treatment groups, proving their biosafety (Figure 6E).

**Figure 7:**
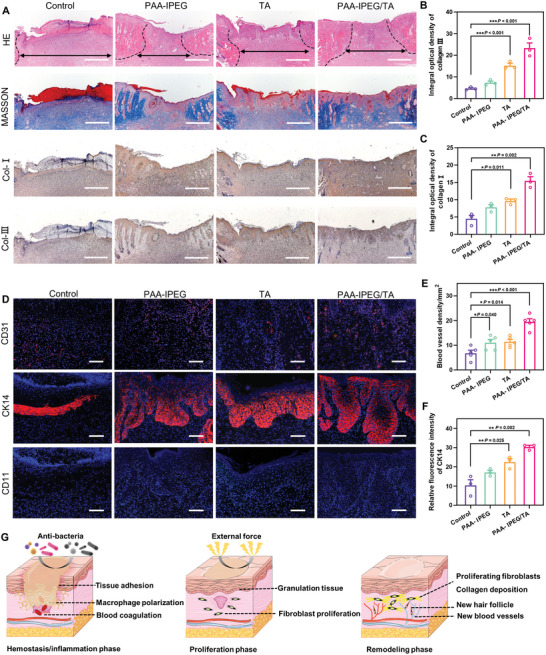
Histological evaluation of wound tissue on day 14. A) Images of H&E‐stained, Masson's trichrome‐stained, and immunostained collagen I and III of skin tissue within the wound area on day 14. Scale bar = 500 µm. B,C) Quantitative results of collagen type I and III on day 14. D) Immunofluorescence images of CD31 (red), CK14 (red), and CD11b (green) in skin tissue within the wound area on day 14. Scale bar = 100 µm. E) Quantitative blood vessel density on day 14. F) Quantitative analysis of the relative fluorescence intensity of CK14 in skin tissue. G) Illustration of the therapeutic activities of the PAA‐IPEG/TA coacervate hydrogels at different stages of wound healing. Statistical significance was expressed as *p < 0.05, **p < 0.01, and ***p < 0.001. Data are means ± S.E.M. (standard error of the mean).

To histologically assess the therapeutic effect and deduce the corresponding mechanism, hematoxylin and eosin (H&E) and Masson's trichrome staining were performed on samples of regenerated skin tissue from wounds collected on day 14 (**Figure** **7**A). It was found that, unlike the treatment groups where wounds displayed significant degrees of closure, the control group showed much less wound healing based on the sizeable remaining subcutaneous distance, parakeratosis, epidermal hyperplasia, and inflammatory infiltration by H&E staining, and disordered deposited collagen fibers and thick residual scabs by Masson staining. The PAA‐IPEG/TA‐treated group had the most intact epidermis, the thickest collagen fiber deposition, and different skin appendages (like hair follicles), demonstrating that the PAA‐IPEG/TA coacervate hydrogels had the best therapeutic efficacy.

Next, immunostaining of type I collagen, type III collagen, CD31, cytokeratin 14 (CK14), and CD11b was performed to assess a variety of important wound healing indicators, including collagen formation, angiogenesis, re‐epithelialization, and inflammatory reaction. Collagen types I and III are the two main components in the dermis, which are important in wound remodeling. Adequate collagen type III is necessary for the reduction of scar tissue formation.^[^ ^67^ ^]^ As shown in Figure 7A, all groups had positive staining of collagen type I and III. The collagen‐related optical density in the PAA‐IPEG/TA‐treated group was significantly higher than that in control groups (*p* < 0.001 for collagen type I and *p* = 0.002 for collagen type III, Figure 7 B,C), suggesting an effective biofunctional activity of PAA‐IPEG/TA in promoting collagen expression and reducing scarring. Angiogenesis is another beneficial event for wound healing since new blood vessels can provide the necessary supply of nutrients and oxygen for tissue reconstruction and metabolic waste transport.^[^ ^68^, ^69^ ^]^ Immunofluorescence staining of CD31 was carried out to assess angiogenesis in the regenerated tissue (Figure 7D). The red fluorescent signal (CD31) of the control group was the lowest. The corresponding quantitative analysis showed that the treatment groups all had significantly higher blood vessel density than the control group, especially the PAA‐IPEG/TA‐treated group (*p* < 0.001, Figure 7E). These findings demonstrate that the PAA‐IPEG/TA coacervate hydrogel can accommodate a favorable environment for promoting wound healing. Since epithelialization is a crucial indicator for complete wound closure, we measured cytokeratin 14 by immunofluorescence staining of CK14, primarily expressed in the hairs and epithelial cells,^[^ ^70^ ^]^ to evaluate the re‐epithelialization (Figure 7D). The corresponding images showed that the wound areas were fully encased in the keratin layer in all treatment groups except in the control group. The relative fluorescence intensity of CK14 (Figure 7F) confirmed the highest keratin expression level in the PAA‐IPEG/TA‐treated group (*p* = 0.002, vs the control group), indicating that the PAA‐IPEG/TA coacervate hydrogel has the best pro‐epithelialization ability. Finally, the inflammatory reaction in the wound area was assessed via immunofluorescent staining of CD11b (Figure 7D). TA, PAA‐IPEG, and PAA‐IPEG/TA shared desirable biocompatibility because no fluorescence among the three groups was detected. These immunostaining results confirm that the PAA‐IPEG/TA coacervate hydrogel can induce no sign of inflammation and accelerate wound recovery by promoting collagen deposition, angiogenesis, and re‐epithelialization.

To systematically validate the preclinical safety of the PAA‐IPEG/TA coacervate hydrogels, we performed in vivo histopathology of the major organs (heart, liver, spleen, lung, and kidney) and hematological examination on day 14. The major organs in the PAA‐IPEG/TA‐treated group showed no apparent tissue damage or pathological changes (Figure S20, Supporting Information), suggesting good histocompatibility. Hematological analysis revealed no significant hematological differences among all groups, suggesting that the PAA‐IPEG/TA coacervate hydrogel did not induce any abnormalities in the hematological system (Figure S21, Supporting Information). These encouraging experimental results demonstrate that the facilely fabricated PAA‐IPEG/TA coacervate hydrogel possesses the necessary biocompatibility and robustly adheres to damaged skin to protect a wound from infection and injury. This dressing also exhibits favorable biofunctions at different stages to promote wound recovery, including blood coagulation in the hemostatic phase, regulating macrophage polarization, promoting fibroblast proliferation in the inflammatory/proliferative phase, and accelerating collagen deposition, angiogenesis, and re‐epithelialization in the remodeling phase (Figure 7G). These results highlight the great potential of the PAA‐IPEG/TG coacervate hydrogel in clinical applications.

## 3 Conclusion

We report a facile method for producing a PAA‐based adhesive coacervate hydrogel from a mixture of PAA‐IPEG and TA. Multiple interactions with both dry and wet substrates stabilize the polyphenol moieties in this coacervate. This structure provides robust adhesion through numerous dynamic hydrogen bonds to achieve wet adhesion and ideal self‐healing after breaks caused by external force or movements. In vitro experiments indicated that the coacervate hydrogel could sustainably release TA, which endowed it with bactericidal activity, and the capacity to promote fibroblast migration and regulate macrophage polarization. We also found that PAA‐IPEG/TA significantly shortened the hemostatic time. In rat experiments, the coacervate hydrogels promoted collagen deposition, angiogenesis, and re‐epithelialization, thus facilitating wound closure and healing. Overall, these results demonstrate that the PAA‐IPEG/TA coacervate hydrogel firmly adheres to wounds to protect them from bacteria and physical trauma and accelerates wound healing. This material can be exploited as a potential wound dressing for clinical applications.

## 4 Experimental Section

### Preparation of PAA‐IPEG Polymer

Acrylic acid and IPEG (*M*_w_: 2400) in molar ratios of 5:1, 10:1, or 20:1 were dissolved in deionized (DI) water to form a homogeneous solution, and then 2 wt% 2,2′‐Azobis(2‐methylpropionamidine) dihydrochloride was added into the solution as the initiator. The polymerization was performed at 50 °C for 8 h to obtain a PAA‐IPEG copolymer solution. The obtained solution was dialyzed (14 000 *M*_w_ cutoff) against DI water for 72 h, refreshed with water every 12 h to remove the unreacted residuals. The dry PAA‐IPEG copolymer was obtained after lyophilization and redissolved in DI water to prepare a polymer solution at a specific concentration. Notably, to optimize the properties, the copolymer was named PAA*x*‐IPEG*y* (see section 2.2), where *x* and *y* represented the molar ratio between PAA and IPEG in PAA‐IPEG.

### Preparation of PAA‐IPEG/TA Coacervate Hydrogel

The PAA‐IPEGA polymer solution (20% w/v) was directly added into the TA solution (50% w/v) with the vortex and subsequently centrifuged for 3 min at 3000 × *g*. The PAA‐IPEG/TA coacervate hydrogels were obtained upon removal of the supernatant and washing it three times with DI water. The PAA‐IPEG/TA coacervate hydrogel powders were obtained after lyophilizing and grinding. To optimize the properties, the coacervate was named PAA‐IPEG*x*/TA*y* (see section 2.2), where *x* and *y* represented the molar ratio between PAA‐IPEG and TA in the coacervate.

### Adhesion Property of PAA‐IPEG/TA Coacervate Hydrogels

The lap‐shear strength test was carried out using the MTS‐E44 universal tester (MTS Systems Co. Ltd., China) with various substrates (20 mm × 25 mm × 2 mm), including iron, glass, and PMMA. Before adhesion, the substrates were cleaned with ethanol and DI water and thoroughly dried at 60 °C for 30 min. Subsequently, one piece of the PAA‐IPEG/TA coacervate (100 mg) adhered to one substrate, and another substrate was placed on the top of the coacervate with an overlapped bonding area of 20 mm × 25 mm. After drying at room temperature for 24 h, each sample was subjected to a lap‐shear test to measure the adhesion strength at the controlled rate 10 mm min^−1^. The lap‐shear adhesion strength was calculated by dividing the maximum failure force by the overlap area, while the shear strength was obtained from the shear stress at the point of detaching (*n* = 3).

Underwater tensile strength tests were also performed using the MTS‐E44 universal tester (MTS Systems Co. Ltd., China) with modifications. A glass tank (300 mm in diameter and 100 mm in height) with DI water was applied to simulate the underwater environment and fixed on the universal tester. A small PMMA cuboid was set at the bottom of the glass tank and ≈50 mg of the PAA‐IPEG/TA coacervate adhered to the upper side of the PMMA cuboid. Subsequently, another PMMA cuboid was pressed on the top of the coacervate under a force of 5 N. After pressure for 30 s, the universal testing machine pulled the upper PMMA cuboid at a speed of 10 mm min^−1^ until the bonding area was broken. The bonding area was always underwater during the stretching process and the tensile strength was calculated based on three duplicates.

The iron substrate was respectively immersed in a neutral aqueous solution (pH = 7), an acidic aqueous solution (pH = 3–6) or an alkaline aqueous solution (pH = 9–11) for underwater adhesion tests. About 50 mg of PAA‐IPEG/TA coacervate adhered to the upper side of the iron substrate. Then, another iron substrate was pressed on top of the coacervate with a force of 5 N for 30 s. Subsequently, each sample was removed from the solution and subjected to a lap shear test to measure the adhesive strength at a controlled rate (10 mm min^−1^). The lap shear bond strength is calculated by dividing the maximum force at failure by the lap area (*n* = 3).

### In Vitro Macrophage Polarization Modulation by PAA‐IPEG/TA Coacervate Hydrogel

The DMEM cell medium was adopted to leach the PAA‐IPEG, TA, and PAA‐IPEG coacervates for 24 h. The leach liquors were used in immunofluorescence staining assays to qualitatively analyze the expression levels of iNOS (M1‐type marker) and CD206 (M2‐type marker). Briefly, RAW 264.7 macrophages were transferred to a 12‐well plate (1 × 10^5^ cells per well) and incubated at 37 °C (5% CO_2_) for 12 h. Then, the macrophages were exposed to LPS for 12 h and incubated with the leach liquor for 24 h. After being fixed by 4% paraformaldehyde and washed by PBS with 0.1% Triton‐X, the macrophages were incubated with the primary rabbit polyclonal antibodies for iNOS and CD206 (Beyotime, Shanghai, China) at 4 °C overnight. Finally, the macrophages were incubated with the goat anti‐rabbit antibodies (FITC and Cy3, Beyotime, Shanghai, China) at room temperature for 1 h. At the same time, DAPI was used to stain the cell nuclei. The stained images were collected via a super‐resolution laser scanning confocal microscope (SRLSCM, Olympus Corporation, FV3000).

### In Vitro Antibacterial Properties of PAA‐IPEG/TA Coacervate Hydrogels

*E. coli*, *S. aureus*, and MRSA were adopted to assess the antibacterial properties of the PAA‐IPEG/TA coacervate hydrogels. Specifically, bacterial suspension was added to a 12‐well plate with a density of 10^6^ CFU per well. The dispersion of PAA‐IPEG, TA, or PAA‐IPEG/TA (1 mg mL^−1^ in PBS) was added to the 12‐well plate with the dosage of 1 mL per well and incubated with the bacteria at 37 °C. After co‐culture for 12 h, 10 µL of bacterial suspension in the well was diluted to 1 mL and then spread in the Luria–Bertani medium to determine the number of bacteria (*n* = 3). Bacteria incubated with PBS were set as the control group. Furthermore, the fluorescent dye PI and SYBR Green I were added to the incubated bacterial suspensions for live/dead staining. After co‐cultured for 30 min, bacteria were observed and imaged by SRLSCM (Olympus Corporation, FV3000). Besides, the bacteria were collected, fixed (2.5% glutaraldehyde), gradient dehydrated by alcohol, air‐dried, and subsequently observed via SEM (Hitachi, 19A11986) and TEM (Hitachi, HT7800).

### In Vivo Antibacterial Properties of PAA‐IPEG/TA Coacervate Hydrogels

Under general anesthesia, a 15 mm back skin incision was made on the dorsal of female Sprague–Dawley rats (8 weeks old) and 100 µL MRSA suspension (10^7^ CFU mL^−1^) was injected into the wound site. The wounds were treated with PAA‐IPEG, TA, or PAA‐IPEG/TA dry powder. Additionally, Tegaderm film was used to cover the treated wounds. At predetermined time points (24 and 48 h), the rats were euthanized and the wound site was observed and imaged. The subcutaneous tissue of the wound area was harvested and homogenized in 1 mL of sterile PBS solution. Then, 100 µL diluted suspension (1000‐fold) was taken and spread on agar plates. After 12 h of incubation, CFUs on each plate were photographed and counted to assess in vivo antimicrobial properties.

### In Vivo Hemostasis of PAA‐IPEG/TA Coacervate Hydrogel

The mouse liver bleeding model and the mouse tail amputation model were used to evaluate the hemostatic ability of the PAA‐IPEG/TA dry powders. Male Kunming mice (8 weeks old) with a weight of ≈40 g were used to establish a model of liver hemorrhage. First, mice were anesthetized by 8% chloral hydrate solution and then exposed to the liver via thoracotomy. Subsequently, the pre‐weighed clean filter paper was placed under the liver and a 12‐gauge needle was applied to make a 5 mm deep round hole in the liver to induce bleeding. Meanwhile, 40 mg of the PAA‐IPEG, TA, or PAA‐IPEG/TA powders were sprayed on the bleeding site to assess the hemostatic ability. Mice without treatment were set as the control group. The weight of the filter paper and the hemostasis time was recorded when the liver stopped bleeding. The blood loss can be calculated based on the weight variation of the filter paper (*n* = 3).

FVB mice weighing ≈20 g were applied in the mouse tail amputation experiments. After being anesthetized, the mouse tail with a 4 cm length was cut off from the end and pre‐weighed clean filter paper was placed under the tail. Simultaneously, 40 mg of the PAA‐IPEG, TA, or PAA‐IPEG/TA powders were respectively sprayed on the wound to evaluate the hemostatic ability. Mice without treatment were set as the control group. The weight of the filter paper and the hemostatic time were recorded when the tail stopped bleeding. The blood loss can be calculated based on the weight variation of the filter paper (*n* = 5).

### In Vivo Wound Healing Evaluation of PAA‐IPEG/TA Coacervate Hydrogel

Female Sprague–Dawley rats (8 weeks old) were used to evaluate the wound healing property. Briefly, a full‐thickness cutaneous wound with a diameter of 10 mm was created on the dorsal of each anesthetized rat and the injury was treated with the PAA‐IPEG, TA, or PAA‐IPEG/TA dry powders. Additionally, a Tegaderm film was applied to cover the treated damage. Subsequently, rats were kept individually with food and water in a specific pathogen‐free (SPF) animal room. The weight of each rat was recorded every day for the following 14 days, and the wound area was observed and captured by a digital camera at predetermined time points. The rats treated only by Tegaderm film were set as the control group. Every group possessed five rats. The wound closure degree can be calculated based on the traces of wound areas changed over time.

When the wound healing experiments were finished on day 14, the wound site and adjacent normal skins were harvested from the euthanized rats and then sectioned for H&E staining, Masson's trichrome staining, and immunostaining (collagens I and III, CD31, K14, and CD11b) to further assess the wound healing. Meanwhile, the blood of rats was also collected for hematological analysis via the hematology analyzer BC‐2800 VET (Mindray, Guangdong, China).

### Statistical Analysis

Statistical analysis was conducted using a Student's *t*‐test or ANOVA test and followed by a Bonferroni post hoc test (GraphPad Prism). Data were presented as mean ± standard error of the mean (S.E.M.). Statistical significance was expressed by *p < 0.05, **p < 0.01, and ***p < 0.001.

## Conflict of Interest

The authors declare no conflict of interest.

## Supporting information

Supporting Information

Supplemental Video 1

Supplemental Video 2

Supplemental Video 3

Supplemental Video 4

Supplemental Video 5

## Data Availability

The data that support the findings of this study are available from the corresponding author upon reasonable request.
