# Supplementary material for: Correction to “Bioinspired Polyacrylic Acid‐Based Dressing: Wet Adhesive, Self‐Healing, and Multi‐Biofunctional Coacervate Hydrogel Accelerates Wound Healing”
Source: Adv Sci (Weinh). 2026 Jul 3;13(42):e76448. doi: 10.1002/advs.76448 (PMC13334589; doi:10.1002/advs.76448)
Supplement: Supplementary file 1 — Supporting File: advs76448‐sup‐0001‐SuppMat.docx. [file ADVS-13-e76448-s001.docx]

**Supporting Information**

**Bioinspired polyacrylic acid-Based Dressing: Wet Adhesive, Self-Healing and Multi-Biofunctional Coacervate Hydrogel Accelerates Wound Healing**

*Lingshuang Wang, Lian Duan, Ga Liu, Jianfeng Sun, Mohammad-Ali Shahbazi, Subhas C. Kundu, Rui L. Reis, Bo Xiao* and Xiao Yang**

**Experimental Section**

*Materials and Animals*: IPEG was provided by Dynamic (Jiangsu, China), Tannic acid was purchased from Macklin (Shanghai, China). All biological dyes and antibodies reagents were from Beyotime (Shanghai, China), unless otherwise stated. All chemical reagents were bought from Aladin Reagent (Shanghai, China), unless otherwise stated..

Female Sprague-Dawley rats (aged 8 weeks, 250 ± 10 g), male FVB mice (aged 8 weeks, 20 ± 2 g) and male Kunming mice (aged 8 weeks, 30 ± 3 g) were provided by Ensiweier (Chongqing, China). All animal protocols in this study were approved by the Institutional Animal Care and Use Committee of Southwest University (No. IACUC-20211120-05).

*Characterization of PAA-IPEG/TA Cacervate Hydrogel*: The FTIR spectrum were recorded by a Nicolet iS10 spectrometer (Thermo Fisher Scientific, USA) with the wavenumber ranging from 4000 to 400 cm^-1^. Samples for NMR measurements were dissolved in C_2_D_6_SO and the spectrum were recorded by NMR spectrometer (Brooke, USA). The cross-section of the lyophilized specimens was sprayed by gold and the morphologies of the samples were examined by a field emission SEM (FEI, USA). Rheological property of PAA-IPEG/TA coacervate hydrogel was measured by a MCR 302 rheometer (Anton Paar, Austria). The oscillatory frequency sweep tests were carried out from 0.1 to 10 Hz at 25℃. Self-healing behavior was tested by rheological thixotropy tests. Amplitude oscillation strain was switched from small strain (1.0%, 60s) to large strain (400%, 60s) and 5 cycles were carried out. The shear stress-strain characterization of PAA-IPEG/TA coacervate hydrogels was also measured by an MCR 302 rheometer (Anton Paar, Austria). Tests were conducted at 25°C with frequency sweep strains from 0.1% to 200%. All the measurements were repeated three times.

*In Vitro Cytotoxic Evaluation of PAA-IPEG/TA Coacervate Hydrogel*: L929 cells were plated into 96-well plate (1 × 10^4^ cells per well) and incubated at 37℃ (5% CO_2_) for 12 h. TA, PAA-IPEG, PAA-IPEG/TA (10 μg) were immersed in 1640 cell medium for 24 h to prepare the leach liquors. Then, the leach liquors were introduced to the cells and incubated for another 24, 48 and 72 h. The cell growth and cell vitality were evaluated by the MTT method. Additionally, the cells were dyed with Calcein AM/PI and subsequently observed by Super Resolution Laser Scanning Confocal Microscope (Olympus Corporation, Japan).

*In Vitro Promotion of Cell Migration by PAA-IPEG/TA Coacervate Hydrogel*: L929 cells were plated into 6-well plate (8 × 10^5^ cells per well) and incubated at 37℃ (5% CO_2_) for 12 h. TA, PAA-IPEG, PAA-IPEG/TA (10 μg) were immersed in 1640 cell medium for 24 h to prepare the leach liquors. Then, the cells were scratched by a pipette tip. The leach liquors were introduced to the cells and incubated for predetermined time points. The cells were imaged using inverted optical microscopy at 0, 24, 48 and 72 h, respectively. The areas of the scratch were evaluated by the Image J for quantitative analysis (n=3).

*In Vitro Blood-Clotting Performance of PAA-IPEG/TA Coacervate Hydrogel*: Heparinized mouse blood (500 µL) was mixed with PAA-IPEG, TA and PAA-IPEG/TA powder (100 mg) in a test tube, respectively. Subsequently, the test tube was inverted to observe whether the mixtures could stand its weight. To investigate the *in vitro* blood clotting time, 10 mg PAA-IPEG,TA and PAA-IPEG/TA powder were added to consecutive wells of 96-well plate containing 50 µL blood. At preseted time points, each well was softly washed with PBS to completely remove unclotted blood. The time to form a homogeneous and stable clot in the wells was defined as the clotting time.

*Blood Cell Adhesion of PAA-IPEG/TA Coacervate Hydrogel*: Whole blood was respectively dropped onto PAA-IPEG, TA and PAA-IPEG/TA and placed at 37°C for 5 min. Then, the samples were washed by PBS, fixed by 2.5% glutaraldehyde, gradiently dehydrated by ethanol solution and dried at 37°C. The adhered blood cells were observed by SEM (FEI, USA).


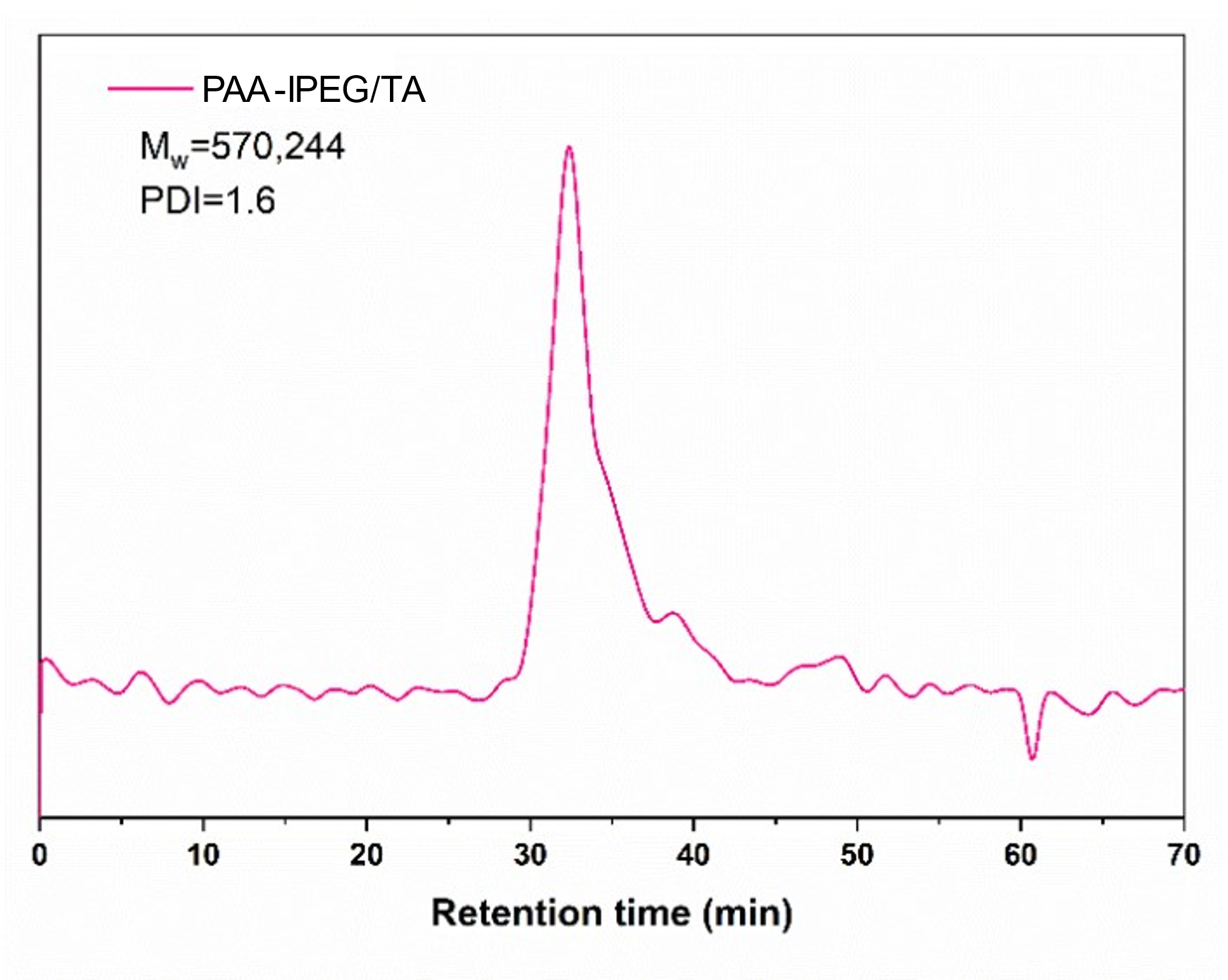
**Figure S1.** GPC curve of PAA-IPEG.

**Figure S2.** Photographs of PAA-IPEG/TA coacervate hydrogel before or after immersing in 6 mol/L urea solution for 24 hours.
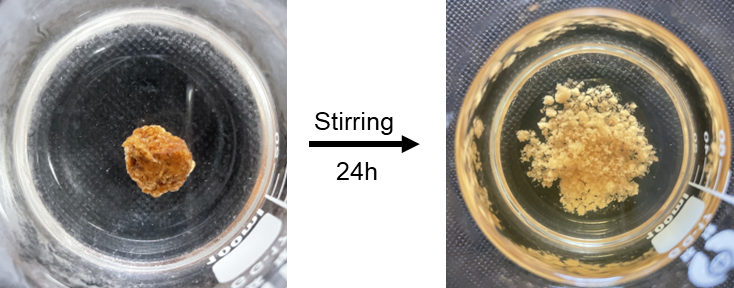


**
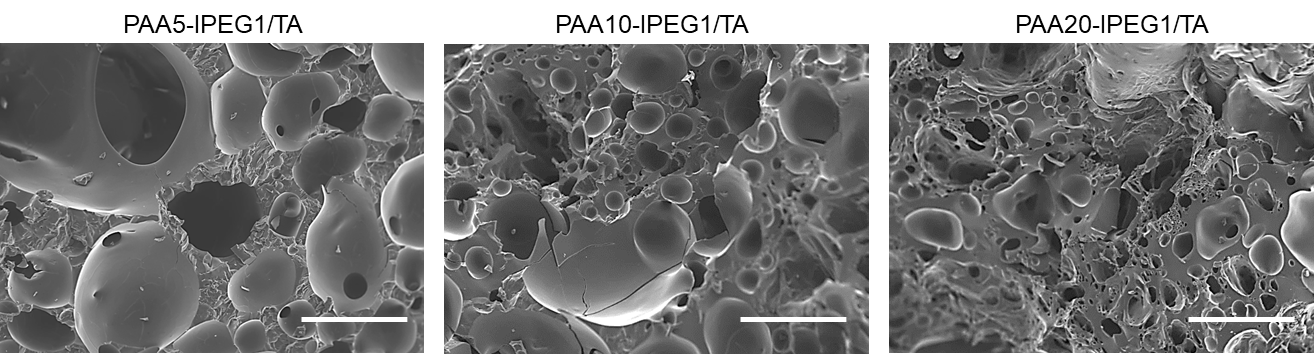
**

**Figure S3.** SEM images of the freeze-dried PAA5-IPEG1/TA, PAA10- IPEG 1/TA and PAA20- IPEG 1/TA coacervate hydrogels. Scale bars = 500 μm.

**
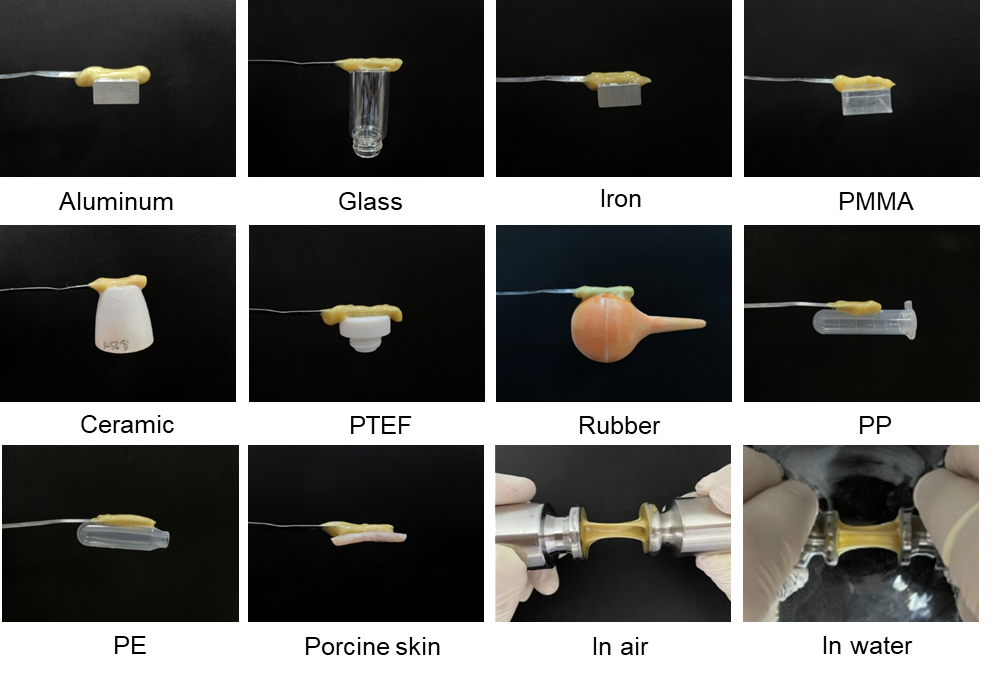
Figure S4.** Demonstration of the adhesiveness of PAA-IPEG/TA coacervate hydrogel to various materials.

**Figure S5.** Photographs of PAA-IPEG/TA coacervate hydrogel adhered to various substrates underwater.
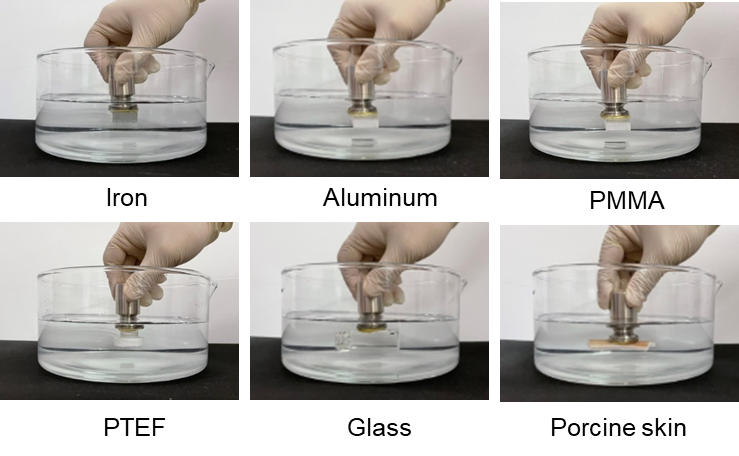


**Figure S6.** Demonstration of the adhesiveness of PAA-IPEG/TA coacervate hydrogel. The PAA-IPEG/TA coacervate hydrogel still adhered to the Poly tetra fluoroethylene plate surface after vigorous water flushing.
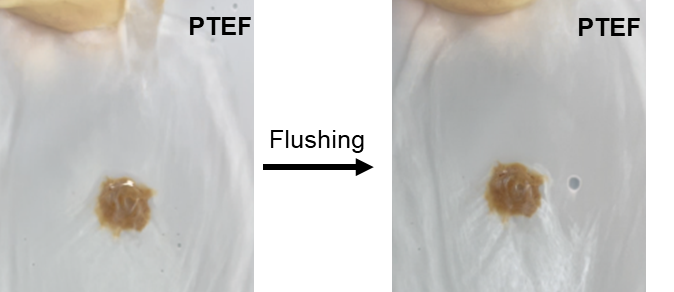


**Figure S7.** Photographs of PAA-IPEG/TA coacervate hydrogel adhered to porcine skin in rotating disc experiments at different time points and the corresponding results (n=3).


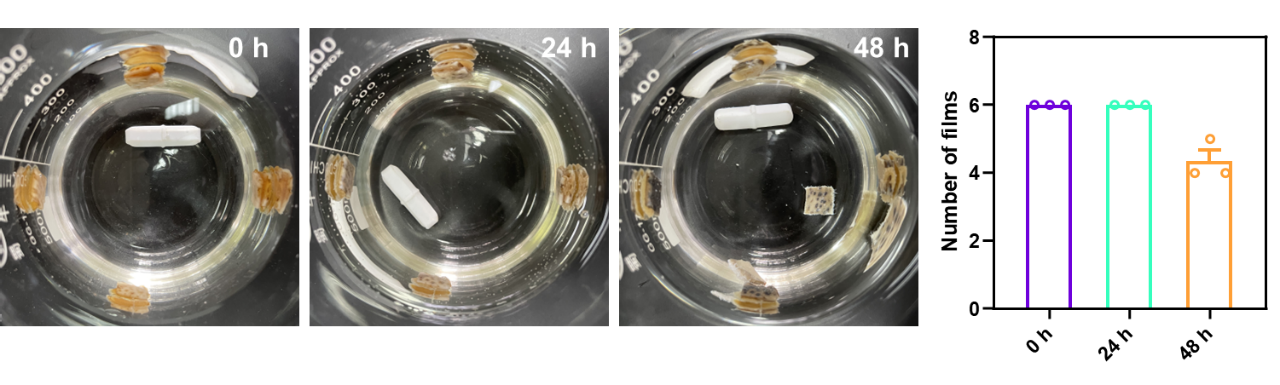


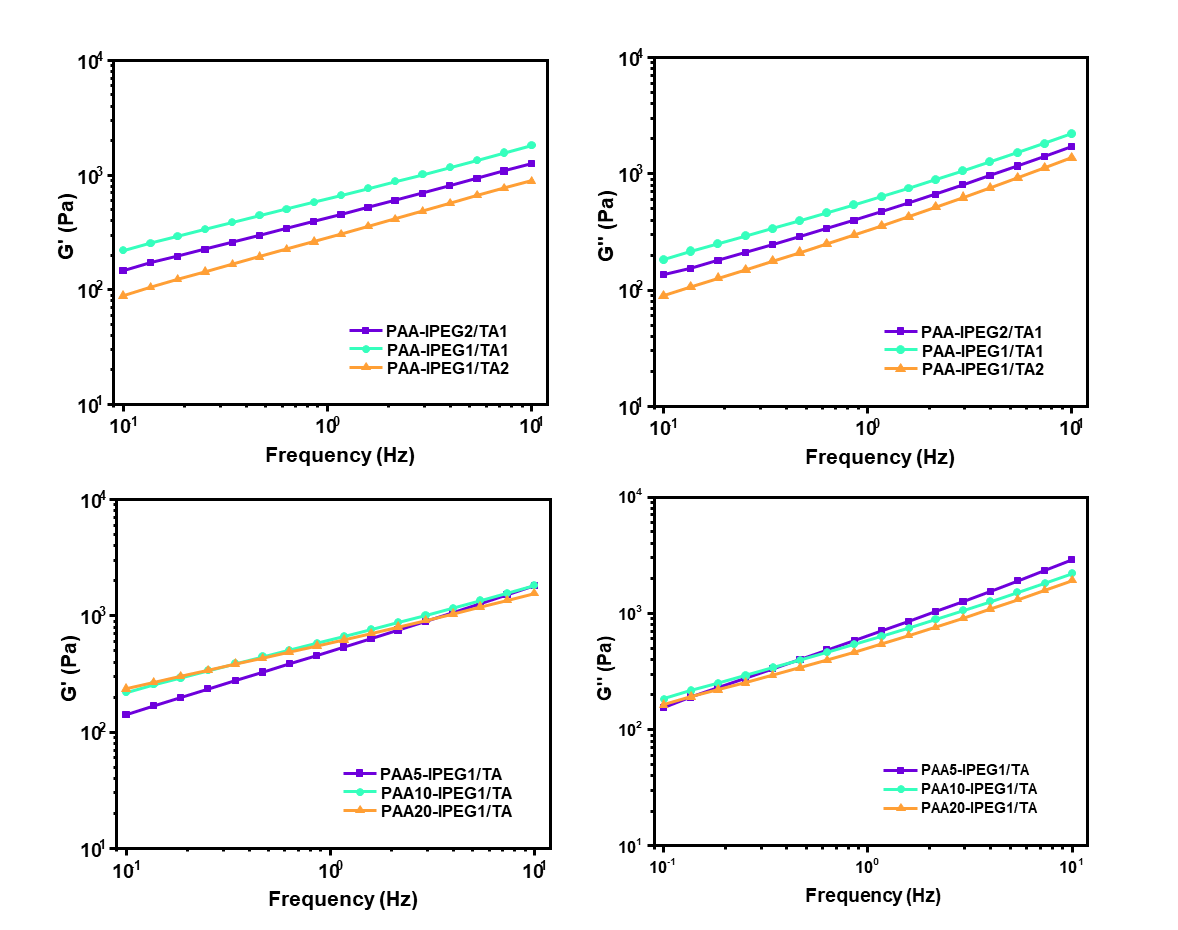


**Figure S8.** The elastic and viscous modulus curves of PAA-IPEG/TA coacervate hydrogels with different ratio of IPEG or TA.


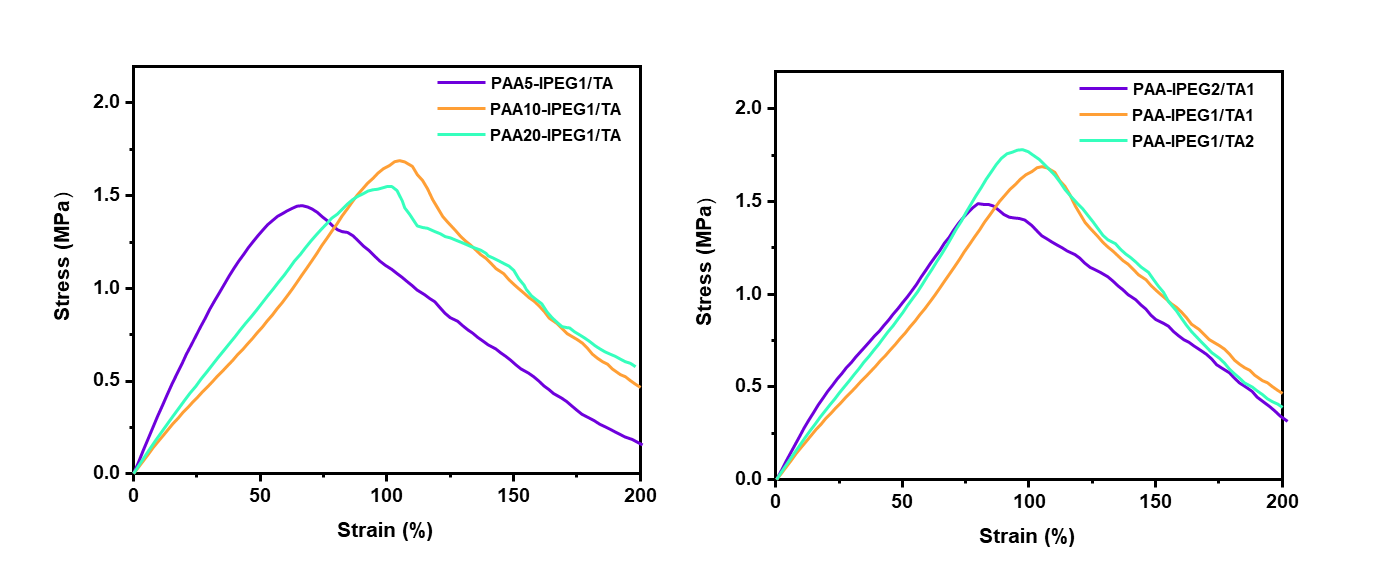


**Figure S9.** The shear stress-strain curves of PAA-IPEG/TA coacervate hydrogels with different composition proportion.


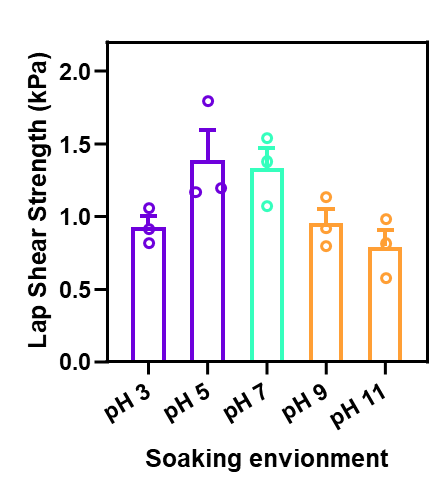
**Figure S10.** Lap-shear adhesive strength of PAA-IPEG/TA coacervate hydrogels on iron substrates at different pH values.

**Figure S11.** Rheological thixotropy of PAA-IPEG/TA coacervate hydrogels with alternate strains switched from 1% to 400%.
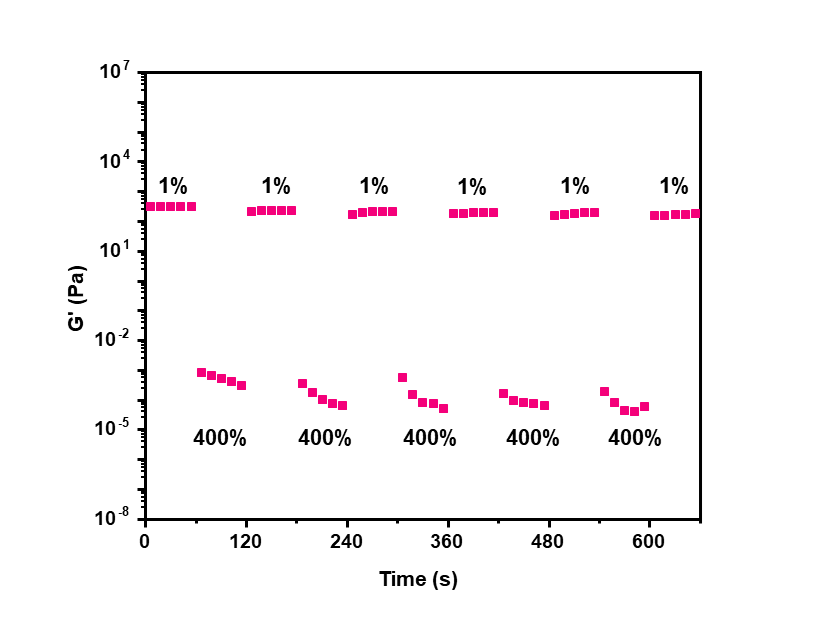


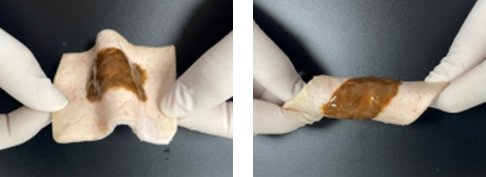
**Figure S12.** Photographs of PAA-IPEG/TA coacervate hydrogel robustly adhered to porcine skin with bending (left) and distorting (right).

**
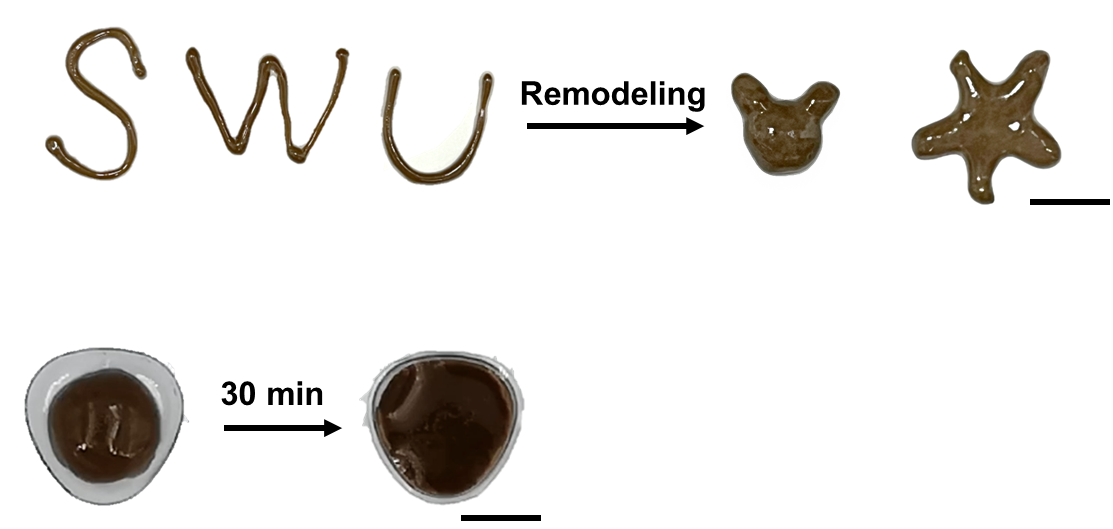
**

**Figure S13.** The photograph of PAA-IPEG/TA coacervate hydrogels with different shapes, including stars, rabbits and Southwestern University logo, to demonstrate the shapeability. Scale bars = 1 cm. **Figure S14.** Photographs of reformed PAA-IPEG/TA coacervate hydrogel withstand 200 g weight after sticking to metal (left), and 500 g weight after curing for 2h
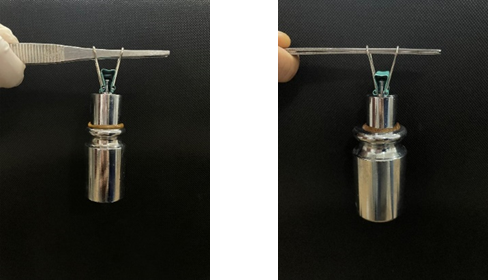
 (right).

**
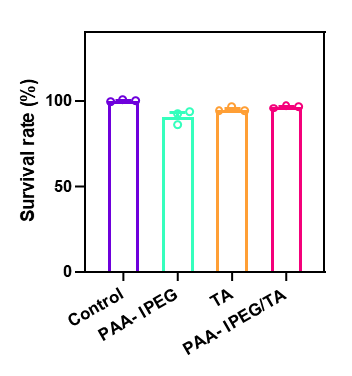
**

**Figure S15.** L929 cell viability after incubating with different leach liquors for 24 (n=3).

**
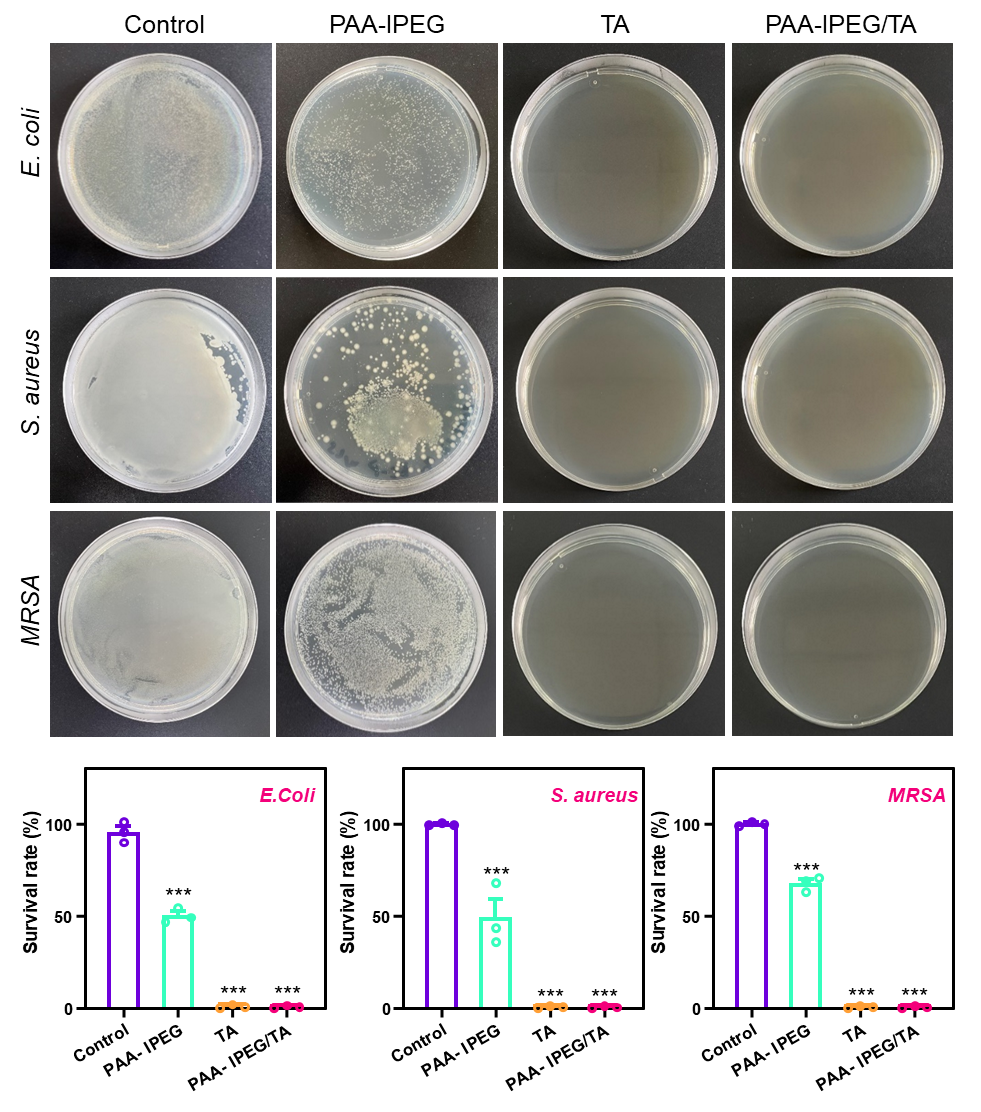
**

**Figure S16.** Photographs of colonies of *S. aureus*, *E. coli* and *MRSA* after incubating with PAA-IPEG, TA and PAA-IPEG/TA coacervate hydrogel for 6 h (n = 3) and the corresponding statistical data.

**Figure S17.** Photographs of the blood gelation process with the addition of PAA-IPEG/TA powder. Liquid blood immediately (within seconds) transformed into a gel state when PAA-IPEG/TA powder was added.


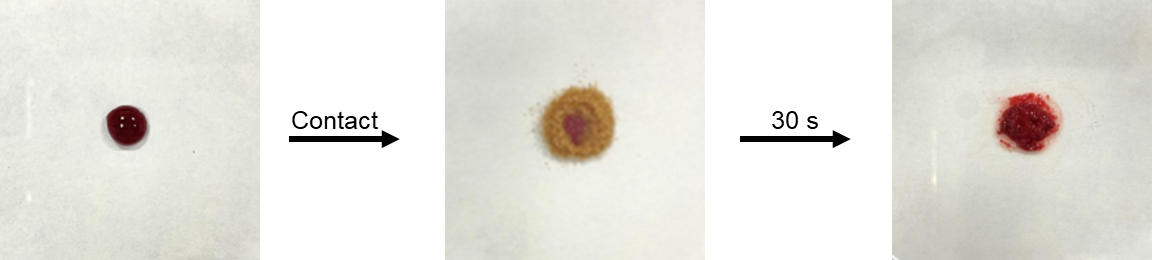


**
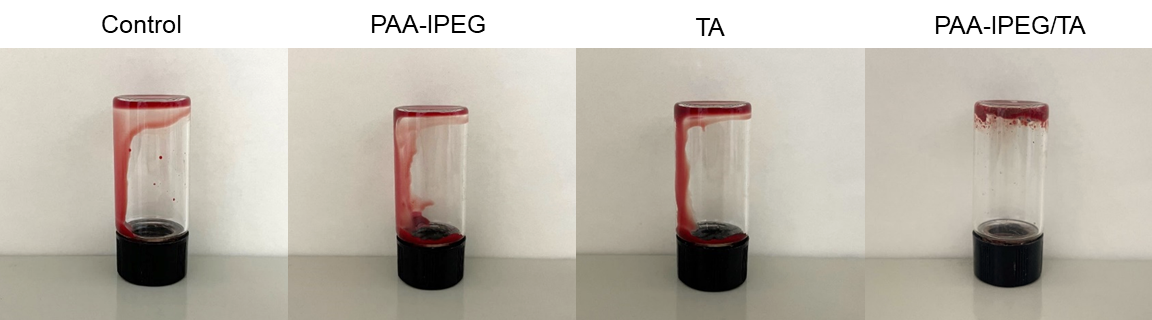
**

**Figure S18.** Photographs of the blood coagulation effect *in vitro*.

**
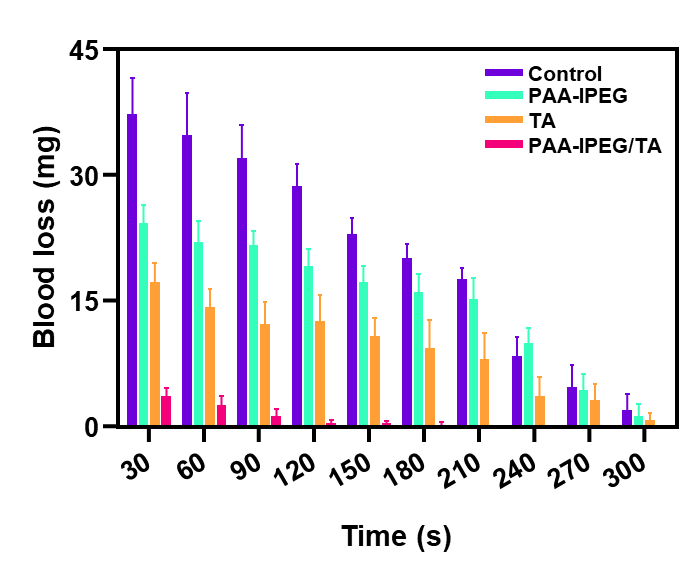
**

**Figure S19.** Blood loss weights in each unit time (30s) during the hemostatic process *in vivo* (n=5).


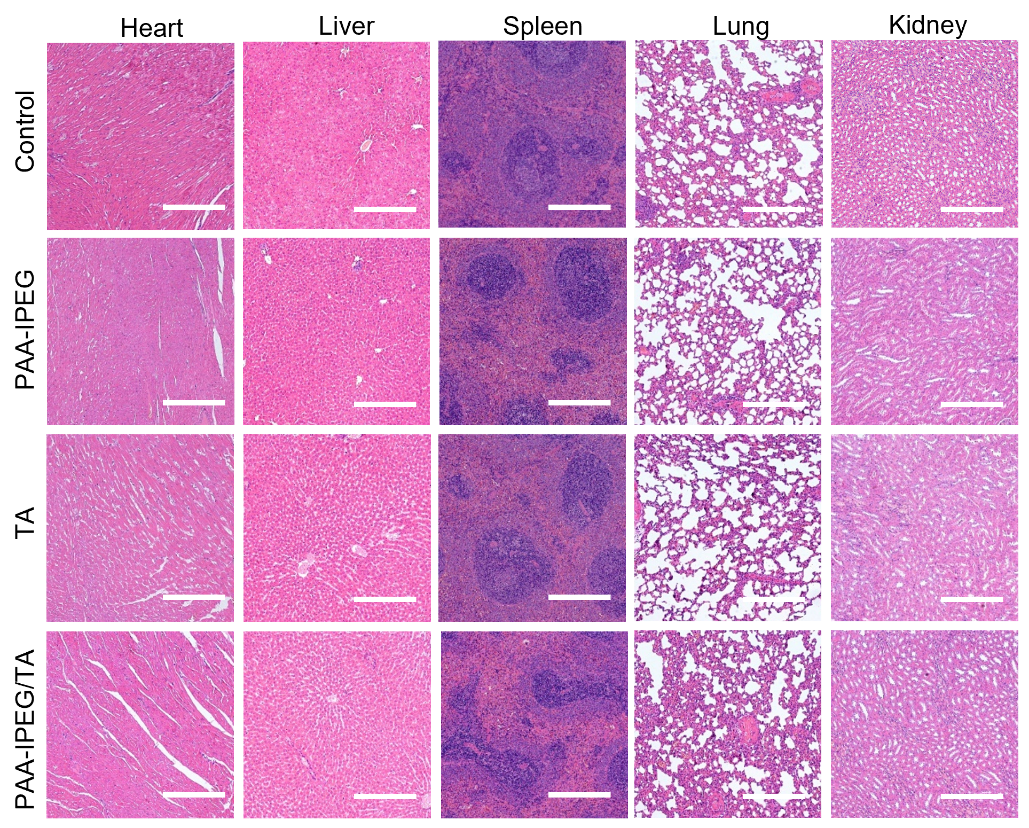


**Figure S20.** H&E staining of major organs (heart, liver, spleen, lungs and kidneys) of rats on day 14 in wound healing experiments. Scale bar = 200 µm.


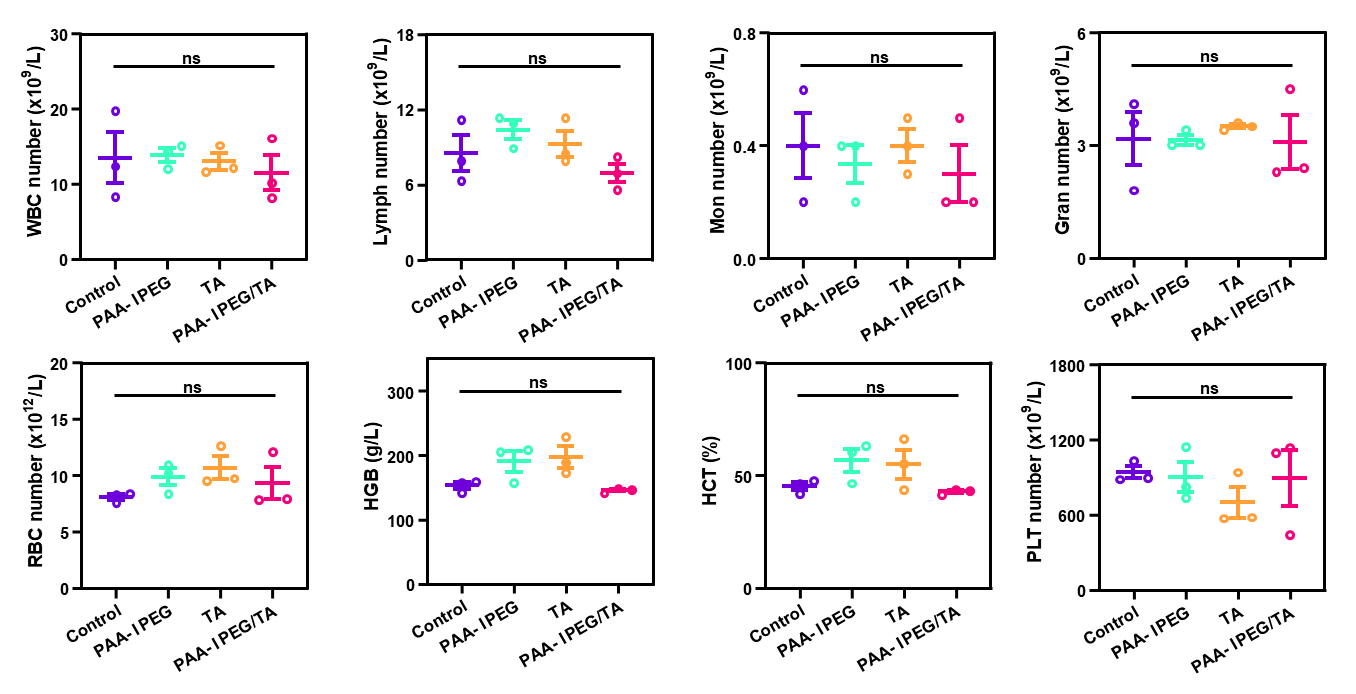


**Figure S21.** Hematological examinations about WBC (red blood cell count), Lymph (lymphocyte), Mon (monocyte), Gran (neutrophil), RBC (white blood cell count), HGB (hemoglobin), HCT (red blood cell-specific volume) and PLT (platelet count) for rats on day 14 in wound healing experiments.
